# Synthesis, DFT and photophysical studies of a new thiophene-fused naphthalene chalcone

**DOI:** 10.1038/s41598-026-49500-4

**Published:** 2026-04-21

**Authors:** M. Yogeesh, Navami Prabhu, Nitinkumar S. Shetty, Rajeev K. Sinha

**Affiliations:** 1 Manipal Institute of Technology, Manipal Academy of Higher Education, Manipal, India; 2 Department of Physics, Birla Institute of Technology, Mesra, Ranchi, 835215 Jharkhand India

**Keywords:** Chalcone, Aldol condensation, Fluorescence, DFT, Chemistry, Materials science, Optics and photonics, Physics

## Abstract

In the present study, we describe the synthesis of a new thiophene-substituted chalcone, namely (*E*)-3-(5-methylthiophen-2-yl)-1-(6-methoxynaphthalen-2-yl)prop-2-en-1-one (P1), through a well-known base-catalysed aldol condensation reaction. The molecular structure of P1 was analysed using a spectroscopic technique, including FTIR, ¹H and ¹³C nuclear magnetic resonance, and mass spectrometry. Further examination of the optical characteristics was conducted using ultraviolet–visible (UV–Vis) and photoluminescence (PL) spectroscopy. UV–Vis spectra of P1, studied using a different solvent, displayed two sets of absorption bands near 290–310 nm and 360–400 nm, which were attributed to π–π* transition, indicating a bathochromic shift. PL measurements revealed a single emission peak centered at 475 nm in solution and a dual-emission profile in the solid state, with maxima at 510 and 610 nm. Furthermore, density functional theory (DFT) calculations at the B3LYP/TZVP level, along with time-dependent DFT (TD-B3LYP/TZVP) for excited-state and absorption studies, were employed to gain deeper insight into the molecular structure and electronic transitions.

**Supplementary Information:**

The online version contains supplementary material available at 10.1038/s41598-026-49500-4.

## 1. Introduction

Chalcones are a class of naturally occurring and synthetically accessible organic compounds that have garnered attention in medicinal and materials chemistry^1^. Structurally, chalcones are characterized by two aromatic rings linked by a three-carbon α, β-unsaturated carbonyl system^2^, and they are found abundantly in nature. Chalcones are typically synthesized through the Claisen-Schmidt condensation^3–5^, an aldol condensation reaction^6–8^, involving an aromatic aldehyde and an aromatic ketone under basic or acidic conditions^9,10^. This method is widely used due to its high yield and ease of introducing structural modifications. The structural flexibility of chalcones enables chemists to modify their pharmacokinetic and pharmacological properties. Chalcone demonstrates diverse bioactive properties. The principal biological activities that have received significant attention include anticancer effects, exemplified by isoliquiritigenin (2′,4′,4-trihydroxychalcone, ISL)^11^, anti-inflammatory properties, as seen in rigid 3,4-dihydroxychalcone (RDHC)^12^, antifungal activity, demonstrated by 2-hydroxychalcone^13^, and antiviral potential, represented by 2′,4′-dihydroxychalcone^14,15^, Alzheimer’s and Parkinson’s^16^. The functional groups, such as hydroxyl, methoxy, and halogen substituents on the aromatic rings, as well as their positions on the chalcone, play a crucial role in influencing these biological activities^15^.

Moreover, chalcones have also attracted attention for their technological applications. Their extended conjugated system makes them valuable in materials science, particularly for developing nonlinear optical (NLO) materials. They can act as second harmonic generation (SHG) materials and have been explored as fluorescent probes for detecting metal ions and biomolecules, highlighting their versatility beyond biomedical applications^17,18^. Chalcones represent a highly versatile and valuable class of compounds in both natural and synthetic chemistry^19^. Their conjugated α, β-unsaturated carbonyl system exhibits intriguing photophysical properties, enabling π–π* and n–π* electronic transitions. As a result, chalcones typically exhibit strong UV–Vis absorption and notable fluorescence, which makes them promising candidates for applications in optical sensors, bioimaging, and photodynamic therapy. Moreover, the nature and position of substituents on the aromatic rings play a crucial role in modulating their absorption and emission spectra as well as their quantum yields^20^.

Thiophene chalcones are a class of heterocyclic compounds synthesized from thiophene-containing ketones and aromatic aldehydes^21^. These derivatives are distinguished by a thiophene ring integrated into the chalcone framework, consisting of two aromatic rings connected by a three-carbon α, β-unsaturated carbonyl moiety^22^. Introducing a thiophene ring, a five-membered heterocycle containing sulfur, significantly improves chalcones’ electronic and pharmacological properties, rendering thiophene-substituted chalcones highly valuable in medicinal and materials chemistry^23^. These findings encouraged us to synthesize a new thiophene-containing chalcone. This study employs density functional theory to investigate the synthesis and photophysical properties, including UV analysis, fluorescence studies, and theoretical calculations.

## 2. Experimental

### 2.1. Materials and methods

All chemicals used for synthesis and characterization were of analytical grade, procured from commercial suppliers, and used without further purification. The progress of the reaction was monitored by thin-layer chromatography (TLC) on silica gel–precoated alumina plates, employing a suitable mobile phase. The melting point was measured by the open capillary method using a Thiele’s tube apparatus.

#### 2.1.1 Analytical and spectroscopic characterization

Solid-state characterization was performed using Attenuated Total Reflectance (ATR) on a Shimadzu FTIR spectrometer, with spectra recorded in the 400–4000 cm⁻¹ range at room temperature, averaging 40 scans at a resolution of 4 cm⁻¹. Nuclear Magnetic Resonance (NMR) spectra were recorded on a Bruker spectrometer using DMSO-d_6_ as the solvent and tetramethylsilane (TMS) as the internal reference. UV–Vis absorption spectra of 0.01 mM solutions in DMF were obtained using a Shimadzu UV-3600 spectrophotometer with quartz cuvettes. Fluorescence measurements were performed in both solid and solution states (0.01 mM in DMSO) at room temperature using a Horiba FluoroMax Plus spectrofluorometer (model Fluromax_Plus_c, serial no. 08446-3324-FM PLUS) using FluorEssence™ software equipped with a 150 W xenon arc lamp. A 390 nm excitation wavelength was used for all fluorescence experiments. The spectral correction procedure was used to account for the instrumental response. A UV -visible spectrum showed absorption maxima near 330 nm at 0.01 mM concentration, and it extended up to 400 nm. An excitation wavelength of 390 nm was set within this band to minimize artifacts and confirm reproducibility. At this concentration, the absorption maximum near 390 nm is less than 0.9 AU, confirming the negligible inner-filter effect. Thus, emission intensity is not distorted by concentration-dependent artifacts. The quantum yield can be calculated using a Horiba Fluorolog QM series with an excitation wavelength of 400 nm at a P1 concentration of 0.01 mM in DMSO. The fluorescence quantum yield (Φf) of compound P1 can be determined by an absolute method using an integrating sphere coated with Spectralon reflective material. No standard reference was needed. The sample was prepared in DMSO (0.01 mM) at 400 nm, and the quantum yield was calculated using the instrument’s quantum yield calculation software. Measurement was done at a single trial, so no error estimates are available.

#### Density functional theory (DFT) calculations

The electronic properties of the molecule in both ground and excited states were investigated through DFT studies. Geometry optimization of the ground state (S_0_) was carried out employing the B3LYP hybrid functional in combination with the TZVP basis set^24^. Harmonic frequency calculations were performed after geometry optimization to confirm that the obtained structure corresponded to the global minimum on the potential energy surface. Ground-state calculations were also performed in a solvent (DMF) environment. Using the optimized ground state structure, vertical excitation calculations were performed to predict the UV-Vis absorption spectrum. For the calculation, the TD-B3LYP functional and TZVP basis set were utilized. The structure was also optimized in excited states with and without a DMSO solvent environment, using the TD-B3LYP/TZVP method. The electrostatic potential (ESP) distribution was calculated to gain insight into the molecule’s electronic behaviour and reactivity. Molecular orbitals, including HOMO and LUMO, were investigated to get an insight into the absorption and emission behaviour of the molecule. All calculations were performed using the Gaussian 09 (Rev. D) program suite^25^.

### Synthesis of (*E*)-3-(5-Methylthiophen-2-yl)-1-(6-methoxynaphthalen-2-yl) prop-2-en-1-one (P1)

In accordance with earlier reports, the target chalcone derivative P1 was prepared through a base-catalyzed Claisen–Schmidt condensation^26^. An ethanolic solution containing equimolar quantities of 2-acetyl-5-methylthiophene (235.7 mg) and 2-methoxy-1-naphthaldehyde (313.04 mg) was treated dropwise with 0.5 mL of 40% aqueous KOH solution under continuous stirring. The reaction mixture was maintained at room temperature for 24 h, and its progress was monitored by thin-layer chromatography (TLC) using a hexane/ethyl acetate mixture (4:1, v/v) as the mobile phase. After completion, the reaction was quenched by the addition of crushed ice, followed by neutralization with dilute hydrochloric acid. The solid product that precipitated was collected by filtration, thoroughly washed with water to remove excess base and inorganic impurities, and dried. Final purification was achieved through recrystallization from ice-cold ethanol, affording the desired chalcone P1 in a pure, crystalline form.

**Fig. 1:**
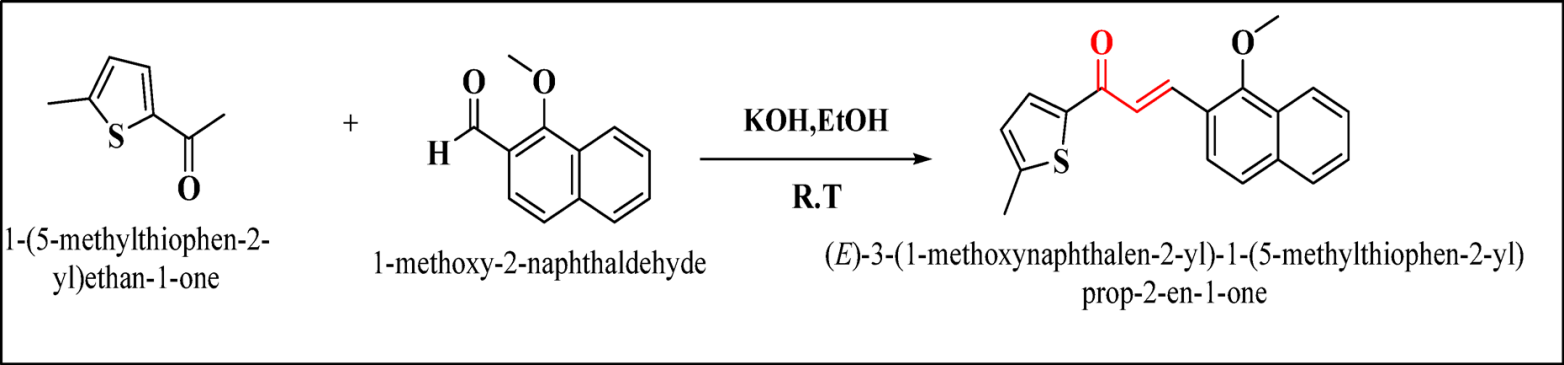
Synthetic route of target compound (E)-3-(1-methoxynaphthalen-2-yl)-1-(5-methylthiophen-2-yl)prop-2-en-1-one(P1) via Claisen-Schmidt condensation reaction of 1-(5-methylthiophen-2-yl)ethan-1-one and 2-methoxy-1-naphthaldehyde.

**Fig. 2:**
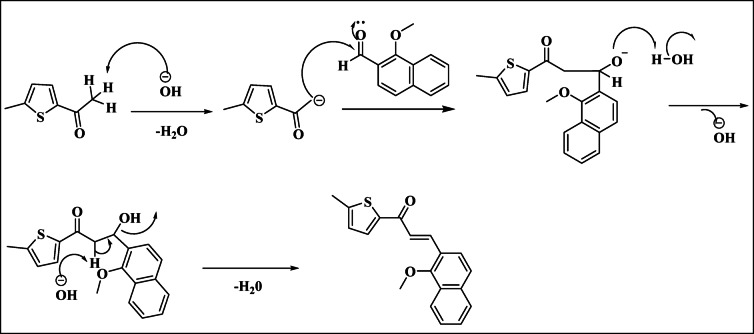
Plausible base-catalysed mechanism for the Claisen Schmidt condensation reaction between the 1-(5-methylthiophen-2-yl)ethan-1-one and base, showing enolate formation, nucleophilic addition to the carbonyl group of 2-methoxy-1-naphthaldehyde, and subsequent dehydration steps leading to the conjugated product P1.

### Analytical and spectral data

Yellow crystalline solid; yield: 90%; melting point: 113–115˚C; FTIR (γ max, cm⁻¹) (Figure S1): 3054 (C–H stretching), 1627 (C=O stretching), 1552 (C=C stretching), 1187 (C–O stretching), 714 (C–S stretching). ¹H NMR (400 MHz, DMSO-d₆, δ, ppm)(Figure S2): δ 8.32–8.29 (d, 1 H, Ar–H, naphthalene), 8.20–8.18 (d, 1 H, Ar–H, naphthalene), 8.08–8.06 (d, 1 H, Ar–H, thiophene), 7.96–7.93 (m, 2 H, Ar–H), 7.86–7.82 (d, 1 H, CH=, vinylic), 7.62–7.42 (m, 6 H, Ar–H), 7.03–7.02 (d, 1 H, CH=, vinylic), 4.07 (s, 3 H, –OCH₃), 2.56 (s, 3 H, –CH₃). ^13^C NMR: (100 MHz, DMSO, δ ppm), δ (Figure S3): 181.95, 157.68, 150.56, 143.76, 135.86, 134.12, 132.72, 129.28, 129.04, 128.33, 128.24, 126.50, 124.36, 123.22, 116.22, 113.88, 56.90, 16.22 ppm. MS (ESI)(Figure S4): m/z 309.1 [M + H]⁺, consistent with the molecular formula C₁₉H₁₆O₂S.

## Results and discussion

### Chemistry

The 2-methylthiophene-substituted chalcone derivative was synthesized via base-catalysed aldol condensation of an equimolar amount of 1-(5-methylthiophene-2-yl)ethane-1-one and 2-methoxy-1-naphthaldehyde in an ethanolic solution, as outlined in Fig. 1. Structure of the synthesized compound confirmed by spectroscopic technique, including FTIR, ¹H NMR, ¹³C NMR, and mass spectrometry. In FTIR spectra, an absorption band at 1627 cm⁻¹ corresponds to the carbonyl group, and absorption bands at 1552 cm⁻¹ and 1187 cm⁻¹ correspond to aromatic C = C stretching and C–O stretching, which is consistent with the presence of an α, β-unsaturated carbonyl system in the thiophene-substituted chalcone. In the ¹H NMR spectrum, a doublet occurs at δ 7.016–7.025 ppm, corresponding to the proton present in the vinylic group which was adjacent to the carbonyl moiety of the compound, followed by another doublet at δ 7.860–7.821 ppm, corresponding to the vinylic proton adjacent to the naphthalene ring. The singlets observed at δ 4.066 ppm and δ 2.558 ppm confirmed the presence of the methoxy and methyl substituents, respectively. The ¹³C NMR spectrum shows several signals that appear in a spectrum that matches the number of carbon atoms present in the synthesized compound, including a carbonyl carbon signal at δ 181.95 ppm, along with aromatic carbon signals, followed by methoxy carbon and a methyl carbon appear at shielded regions at δ 56.90 ppm and δ 16.22 ppm, respectively. Mass spectra confirm the mass of the thiophene-substituted chalcone, which was in close agreement with the calculated molecular weight of C₁₉H₁₆O₂S. The plausible reaction mechanism depicted in Fig. 2 involves, firstly, the formation of a base-catalysed enolate intermediate via the elimination of a water molecule. This enolate then attacks the carbonyl carbon of the aldehyde to form an intermediate alkoxide, which further undergoes intramolecular proton transfer to form a β-hydroxy intermediate. Subsequent dehydration leads to the formation of a conjugated system. Ultimately, cyclization and loss of water yield the desired chalcone.

### DFT calculations: geometry optimization

**Fig. 3:**
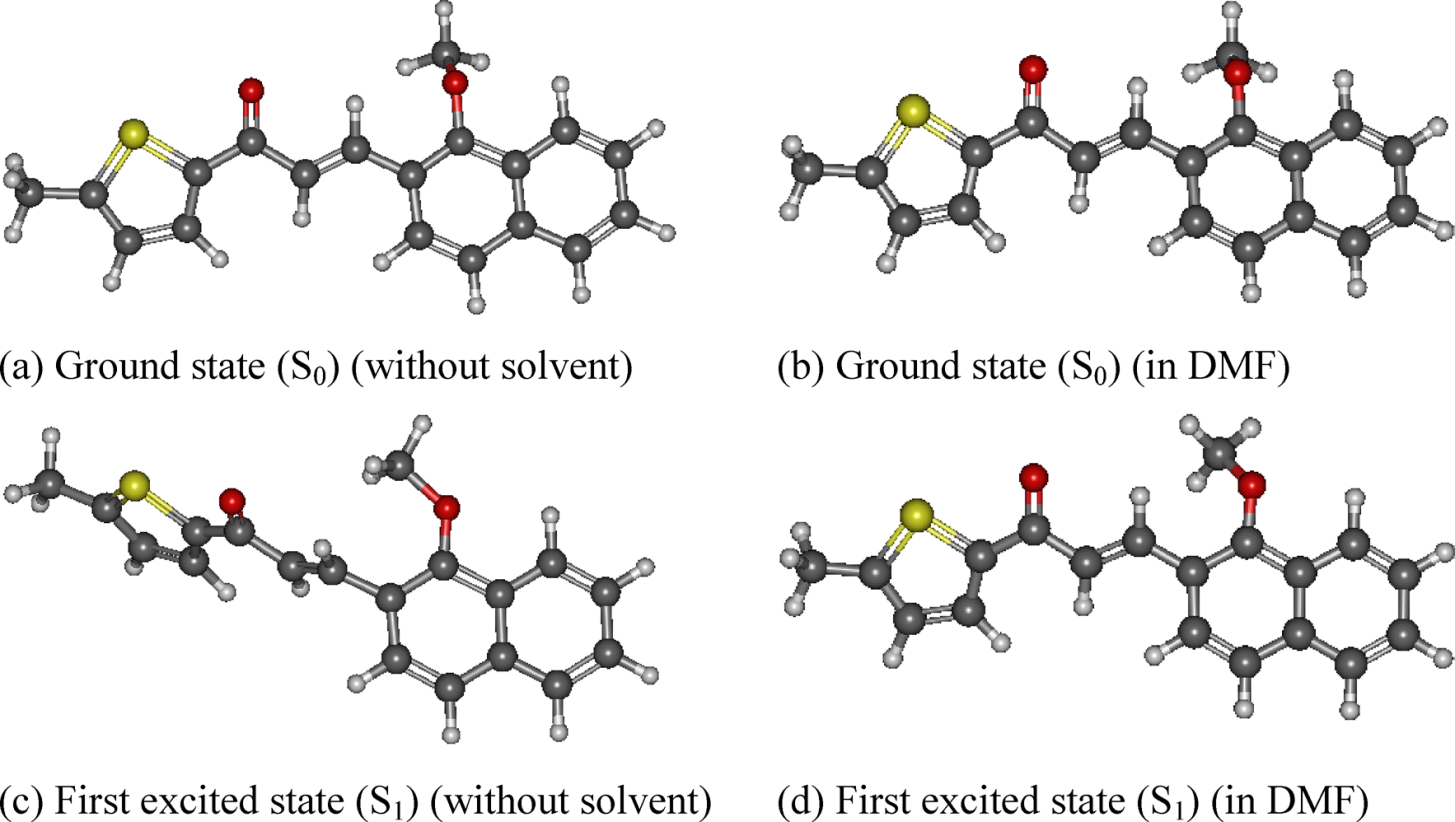
Optimized molecular geometries in the ground state (S₀) and the first excited state (S₁), obtained using B3LYP/TZVP and TD-B3LYP/TZVP methods, respectively. (**a**) Ground state, (S0), (without solvent), (**b**) Ground state, (S0), (in DMF), (**c**) First excited state, (S1), (without solvent), (**d**) First excited state, (S1), (in DMF)

To corroborate the FTIR, NMR, and mass spectrometry results on the structure of the molecule, DFT calculations were also performed. The structures of the molecule in both the ground and excited states were optimized. All ground-state optimizations were followed by frequency calculations and frontier molecular orbital analyses. Figure 3(a) and (b) shows the optimized structure of P1 in the ground (S_0_) state without solvent and in DMF environment. It was observed that the molecule was nearly planar in the S_0_ state without solvent, with the methoxynaphthalene ring ~ 13° out of plane relative to the conjugated enone bridge and the thiophene ring. Under the DMF environment, the angle becomes 9.4°. This almost planar structure enabled effective π-conjugation across the entire donor–π–acceptor system, facilitating efficient electron delocalization. In the absence of solvent, upon excitation to the S_1_ state (Fig. 1c), a noticeable torsional distortion was observed between the central double bond and the adjacent aryl rings, and the methoxynapthalene ring goes out-of-plane by ~ 82^o^ relative to the conjugated enone bridge and the thiophene ring. In contrast, optimization in DMF solvent revealed no significant structural variations. The relative tilt of the napthalene ring in the excited states was 25.5 degrees. The observed much lower twist in the molecule in the S_1_ state in the presence of solvent could be due to the interaction of the solvent molecule with the O-atom of the keto group and the methoxy group with the solvent (polar, aprotic) molecule. A similar effect was observed when the structure was optimized in another polar, aprotic solvent, DMSO. The observed twist upon excitation results in reduced orbital overlap and partial disruption of conjugation, indicating intramolecular charge transfer (ICT). The methoxy group also showed a change in the orientation from out-of-plane to near in-plane after excitation to the S_1_ state, which may influence the charge distribution. These conformational changes were consistent with the nature of the HOMO–LUMO transition and support the ICT character of the excited state.

**Fig. 4:**
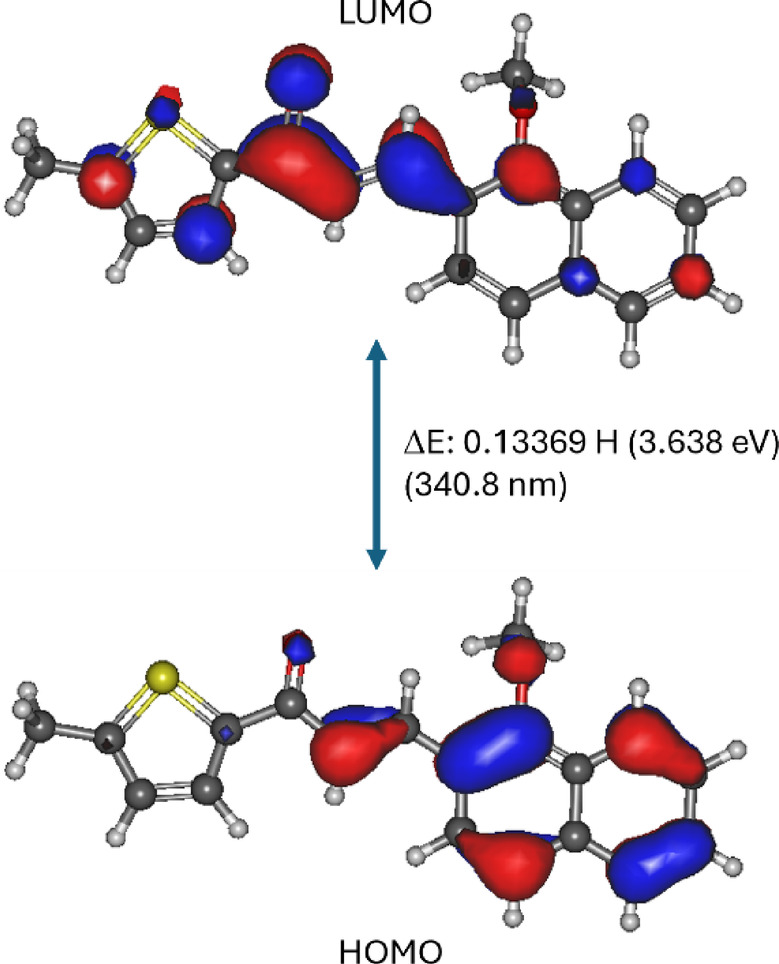
Frontier molecular orbitals (HOMO and LUMO) of compound P1, along with their calculated energy gap (ΔE = 3.638 eV, 340.8 nm).

The observed changes in the structure of the molecule upon excitation to S_1_ state is understood using the frontier molecular orbitals, namely the HOMO and LUMO. The analysis of these frontier orbitals is also important to understand the electronic distribution, optical properties, and general stability of organic molecules. Figure 4 shows the HOMO and LUMO plots with the energy difference between them. As it is evident from the figure, the HOMO has π-symmetry with the electron density largely restricted to the methoxynepthayl ring and adjacent ketoethylenic segment, signifying their role as electron-donating sites. Conversely, the LUMO (π*-symmetry) is predominantly localized on the carbonyl and thiophene moieties, which are electron acceptors, thereby defining a preferred path for electron transfer upon excitation and facilitating an effective intramolecular charge-transfer process. The observed electron densities in HOMO and LUMO explain the observed reorganization in the molecular structure upon electronic excitation. The calculated energy gap between the HOMO and LUMO is found to be 0.13369 H (3.638 eV; ~341 nm), leading to the possibility of absorption close to the UV region (discussed in the next section). In the DMF, the calculated HOMO to LUMO energy gap is 0.12834 H (3.492 eV; ~355 nm).

### Photophysical properties

#### UV-Vis spectral analysis

**Fig. 5:**
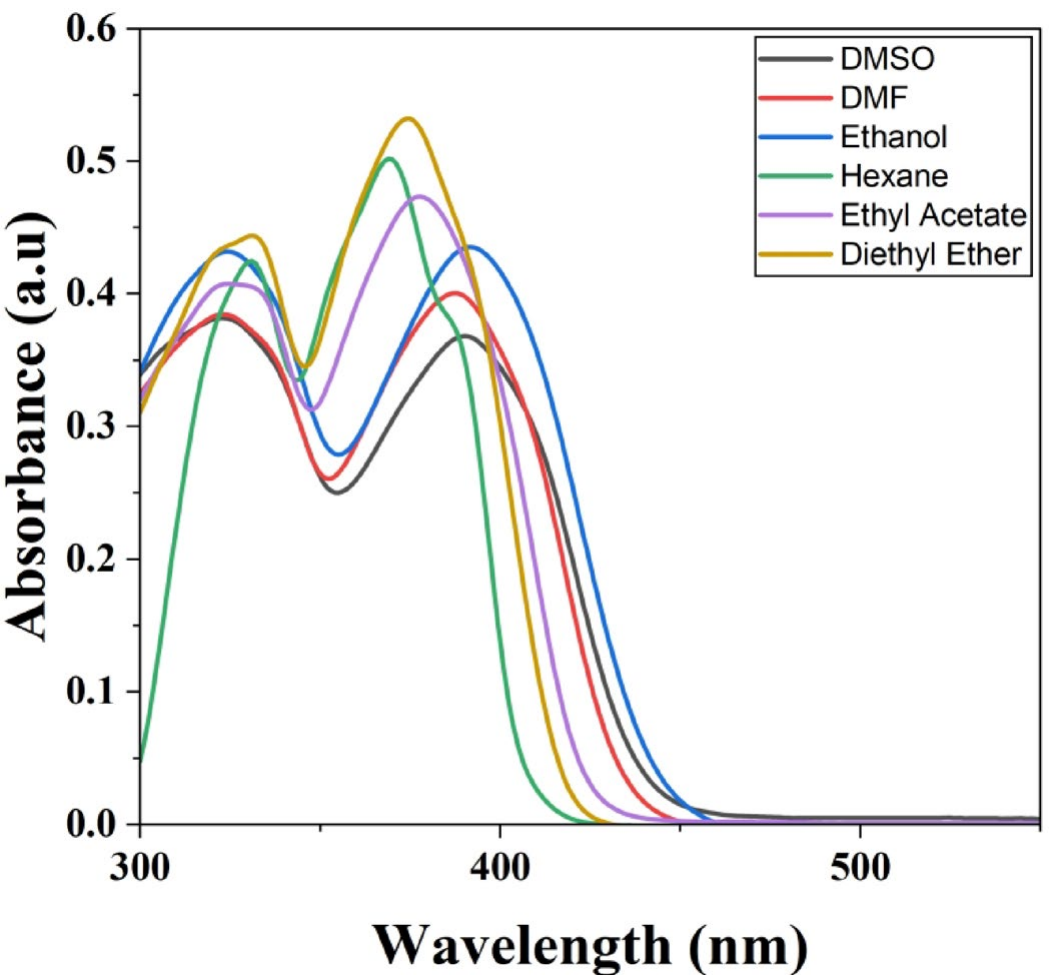
The absorbance spectra of P1 in different solvents, including DMSO, DMF, ethanol, hexane, ethyl acetate, and diethyl ether media at 20 µM concentration, showing dual absorption maximum near 290–310 nm and 360–400 nm.

The UV-Vis absorption spectrum of P1 was studied in different solvents by varying the polarity from DMSO to diethyl hexane, as shown in Fig. 5, revealing two distinct absorption bands. The first band located near the lower wavelength region I, i.e., approximately 290–310 nm, was attributed to π–π* transition present in the conjugated system of aromatic ring or C=C bonds. The second broad peak appeared at near-wavelengths ranging from 360 to 400 nm, corresponding to a π–π* transition in an extended conjugated system or an ICT transition in donor-acceptor substituted molecules.

With increasing solvent polarity, the absorption maximum of the ICT bands shifts from 371 to 396 nm, showing a bathochromic (red) shift. This behaviour indicates enhanced stabilization of the excited state in a polar environment, in agreement with ICT character. The types of these transitions were further supported by theoretically calculated UV-Vis spectra and orbital analysis, indicating dual electronic transition and their sensitivity to solvent polarity.

**Fig. 6:**
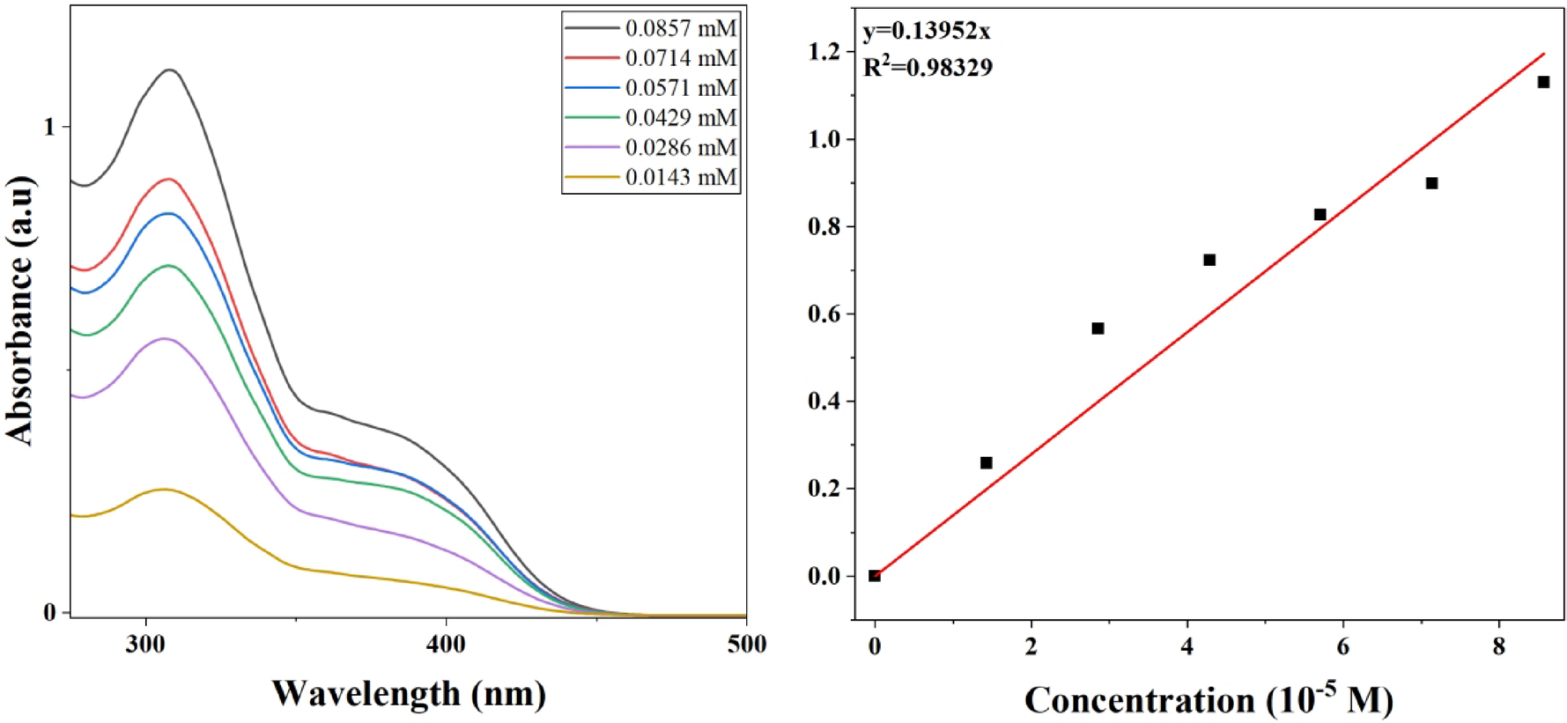
(**a**) The UV–Vis absorption spectra of compound P1 were recorded at concentrations ranging from 0.0143 to 0.0857 mM (DMF), with a maximum absorption peak at 307 nm. (**b**) Plot of maximum absorbance against concentration. The gradient provides a molar extinction coefficient value of ε = 13,952 M^− 1^ cm^− 1^.

The molar extinction coefficient (ε) of the compound was determined at 307 nm using UV-Visible spectroscopy in N, N-dimethylformamide (DMF) as the solvent. Solutions of different concentrations were prepared, and their absorbance values were recorded using a 1 cm quartz cuvette. A linear plot of absorbance versus concentration (Fig. 6b) confirmed that the system obeys the Beer–Lambert law. From the slope of the calibration curve, the molar extinction coefficient was calculated as 1.3952 × 10⁴ M⁻¹ cm⁻¹, indicating a strong electronic transition in the compound.

Using the experimentally determined extinction coefficient (ε = 13,952 M⁻¹ cm⁻¹) and the estimated FWHM of 3342 cm⁻¹, the oscillator strength was found to be 0.21 (calculated using (1)). This experimentally derived value reasonably agrees with the TD-DFT prediction of 0.6659, indicating that the spectroscopic assignment is reliable. Such a difference is well documented in the literature, TD-DFT often overestimates oscillator strengths because it ignores solvent effects and vibronic coupling in solution-state emission measurements. These studies confirm that theoretical values reflect an idealised gas-phase transition, whereas experimental values capture environmental modulation of transition possibilities^27–29^.

1 $${f_{{\mathrm{exp}}}} \approx {\mathrm{4}}.{\mathrm{6}} \times {\mathrm{1}}{0^{ - \,{\mathrm{9}}}} \cdot {{\mathrm{e}}_{{\mathrm{max}}}}*{\text{ FWHM}}$$

**Fig. 7:**
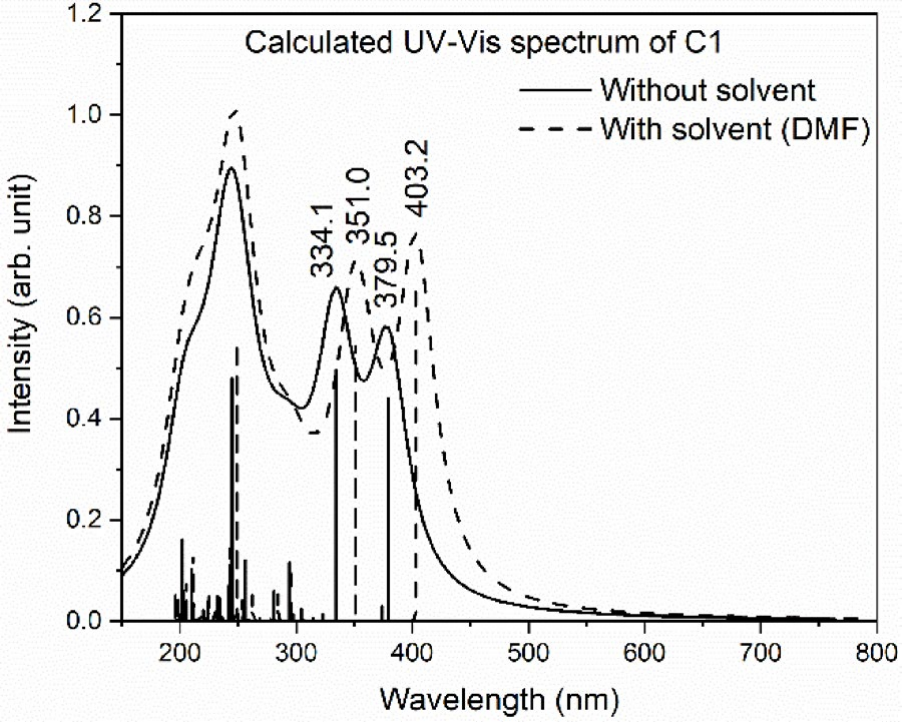
Calculated UV–Vis spectrum of compound P1. Vertical excitation energies were calculated using TD-B3LYP/TZVP theory level.

**Fig. 8:**
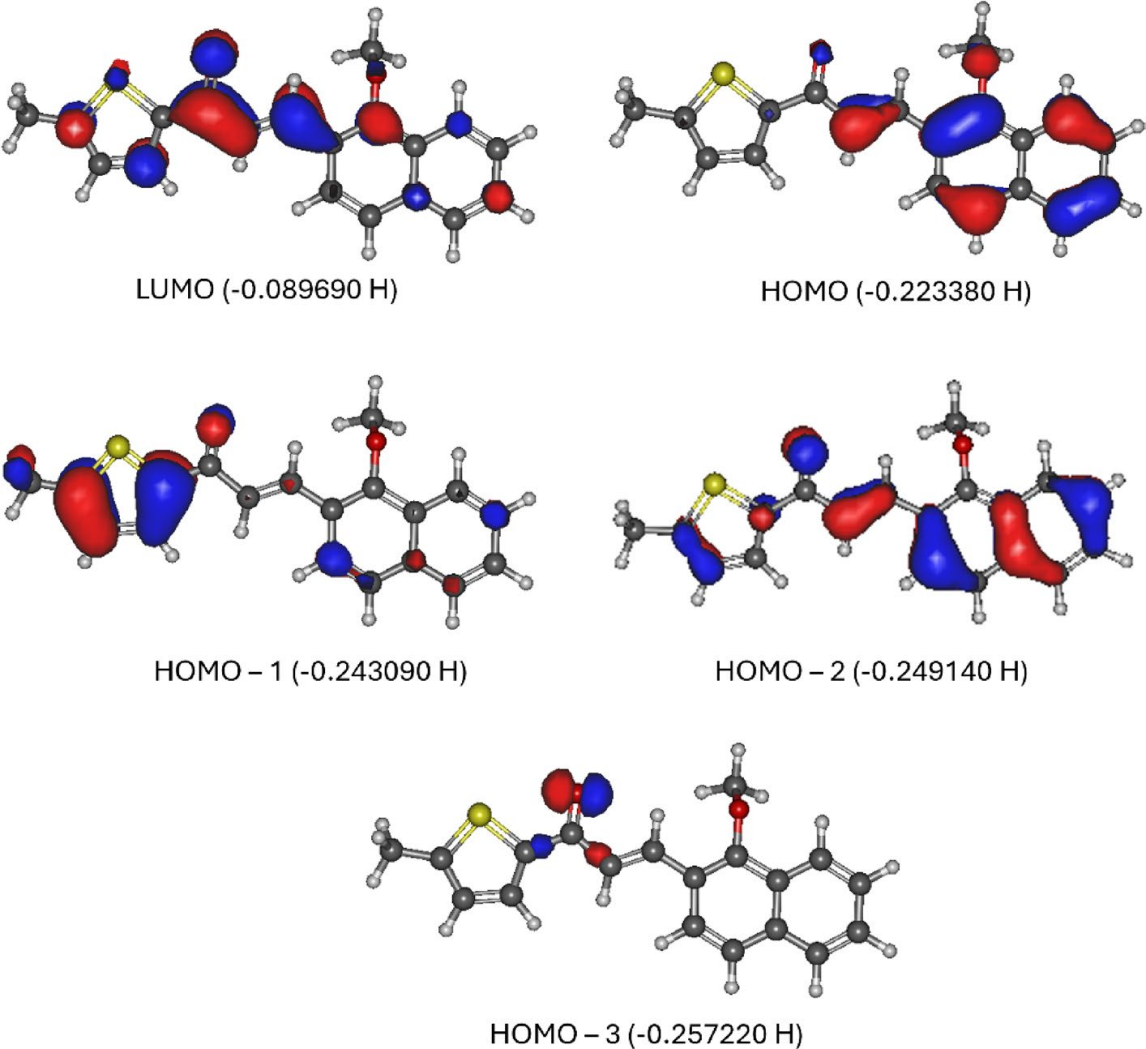
Prominent orbitals involved in the excitation to the first three excited states in P1, showing the main occupied–virtual orbital contributions responsible for the low-energy absorption bands.

**Fig. 9:**
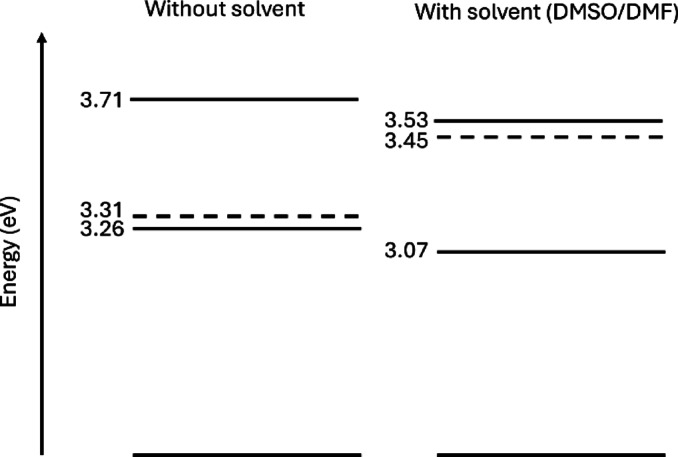
calculated Energy levels of P1 without and with solvent (DMF) environment, illustrating the solvent-induced stabilization and reduction of excitation energies.

Figure 7 shows the calculated UV–Vis spectrum of the molecule P1 with and without DMF solvent. The calculations were performed using TD-B3LYP theory and TZVP basis set. In the calculation, vertical excitation energies of thirty excited states were calculated. Table 1 lists parameters for the first three excited states calculated without solvent. Molecular orbitals with prominent contributions in the first three excited states are shown in Fig. 8. From the orbital plots, it is evident that the HOMO and HOMO-1 have a characteristic of π, whereas HOMO-3 is a lone-pair orbital (n-orbital) localized on oxygen of the keto group, and LUMO shows the characteristic of π*. In the absence of DMF (Fig. 7), the first excited state appears at 379.5 nm with an oscillator strength of 0.4398. The excitation to the S_1_ state is predominantly attributed to the HOMO → LUMO transition, accompanied by a minor contribution from the HOMO–3 → LUMO transition. A better picture of orbitals involved in the transition to the first excited state was also examined using Natural Transition Orbital (NTO) analysis. It was found that the transition is essentially HOMO → LUMO with a coefficient of ~ 0.90. Under the DMF environment, the first excited state corresponds to the HOMO-LUMO orbital transition with absorption maxima at 403.2 nm. Therefore, the characteristic of the first excited state is primarily π→π*. The second excited state is showing primary contribution from n→π* with very low oscillator strength. The third excited state has again appreciable oscillator strength and is categorized as a π→π* transition band. Therefore, the observed bands in the experimental absorption spectrum are primarily due to the π→π*, however, close proximity on the n-orbital may lead to the mixed character of the observed bands. The energy levels obtained with the vertical excitation calculations are shown in Fig. 9. It can be seen that the n→π* transition is in close proximity to the first excited state. In the presence of the solvent, two π→π* transitions get stabilized in energy, whereas the n→π* gets destabilized. In both situations, mixing of n→π* with π→π* may occur.

**Fig. 10:**
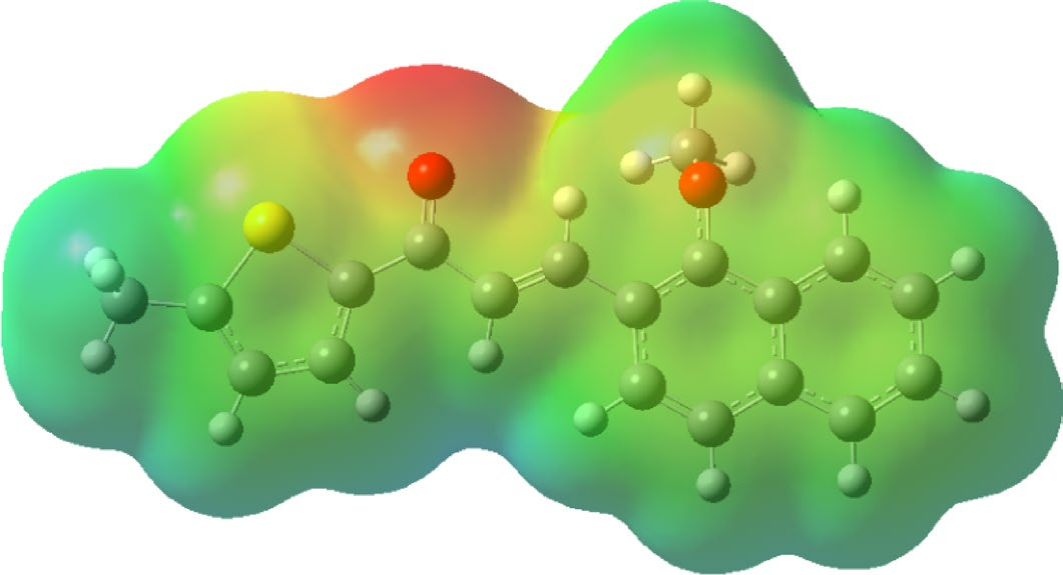
Electrostatic potential energy surface of the molecule P1 showing the spatial distribution of electron-rich and electron-deficient regions.

The calculated bands, without solvent, at 334 and 379.5 nm are red-shifted compared to the observed bands at 303 and 375 nm. This red shift could be due to the use of a moderate basis set, TZVP, which may have underestimated the calculated energies of orbitals. Under the solvent (DMF) environment, the two bands show a further red shift to 351 and 403 nm. This shift could be due to the fact that the use of the solvation model SCRF treats the solvent as a dielectric continuum around the molecule. In contrast, the molecule has a specific affinity at different sites. The electrostatic potential energy surface of the molecule is shown in Fig. 10, where a strong affinity for interaction can be seen at the oxygen atom of the keto group. The solvation model does not consider this specific interaction site, leading to an underestimation of excitation energies, resulting in a redshift of absorption bands.

**Table 1:** Excitation parameters for the first three excited states were obtained with vertical excitation calculation using the TD-B3LYP/TZVP theory level. *HOMO-4 orbital of the molecule in DMF is the same as HOMO-3 without DMF.

	Orbital transition	Co-efficient	Excitation energy (eV)	Oscillator strength
**Without DMF**				
Excited state 1	HOMO-3 → LUMO HOMO → LUMO	0.19439 0.66082	3.2670 (379.51 nm)	0.4398
Excited state 2	HOMO-3 → LUMO HOMO-2 → LUMO HOMO → LUMO	0.65672 0.10764 0.18038	3.3170 (373.79 nm)	0.0290
Excited state 3	HOMO-2 → LUMO HOMO-1 → LUMO HOMO → LUMO + 1 HOMO → LUMO + 2	0.29030 0.60551 0.10427 0.11803	3.7107 (334.12 nm)	0.4968
**With DMF**				
Excited state 1	HOMO → LUMO	0.69706	3.0747 (403.24 nm)	0.6651
Excited state 2	HOMO-4* → LUMO HOMO-1 → LUMO	0.67475 0.11925	3.4518 (359.19 nm)	0.0103
Excited state 3	HOMO-4 → LUMO HOMO-2 → LUMO HOMO-1 → LUMO	0.14159 0.17870 0.65467	3.5320 (351.04 nm)	0.5492

#### Intramolecular charge transfer (ICT)

**Fig. 11:**
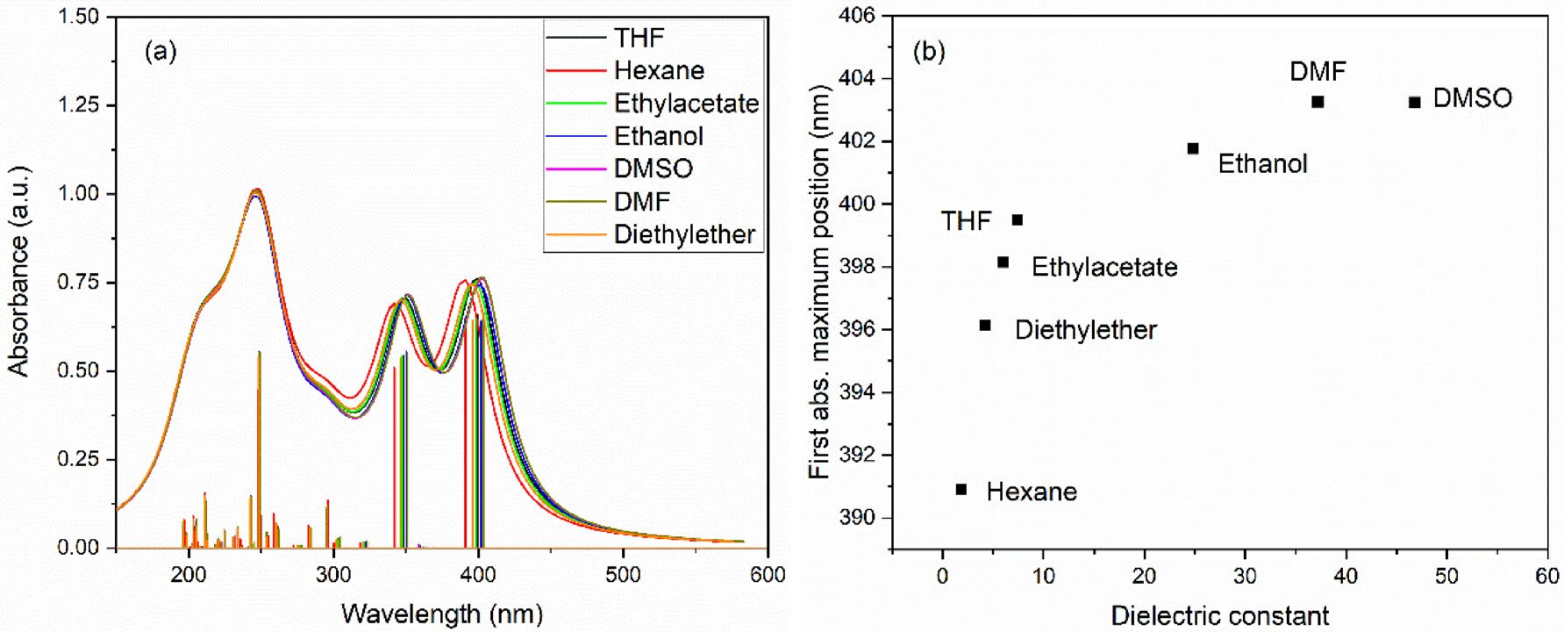
(**a**) Calculated absorption spectra of the P1 molecule in different solvents, (**b**) plot of the first absorption maxima against the dielectric constant of solvents.

The absorption spectra of molecule P1 under different solvent environments, as shown in Fig. 5, clearly indicate a shift in the absorption maxima, centered around 375 nm. To corroborate the experimental observation, the absorption spectra of the molecule were calculated under different solvent environments. Figure 11(a) shows the calculated absorption spectrum. A solvatochromic shift was observed in two bands, centered at ~ 340 and 400 nm. These two bands were assigned as the first and third excited states with the characteristic of π→π*. Figure 11(b) shows the shift in the first excited state maxima against the dielectric constant of the solvent. The observed wavelength shift is consistent with the variation in the dielectric constant. The observed red shift in absorption maxima with an increase in the dielectric constant of the solvent clearly indicates that the charge transfer from π to π* is the principal mechanism in the intramolecular charge transfer.

Natural bond orbital (NBO) was also studied to understand the possibility of ICT. The hyperconjugation energies due to delocalization of electrons from donor orbital to acceptor orbital are investigated^30^. Table 2 lists prominent donor-acceptor orbital interactions along with their corresponding hyperconjugation energies. A complete list of donor-acceptor energy is provided in the supplementary table (ST1). In the Table ST1, Rydberg orbital contributions and antibond-to-antibond interactions are omitted, as they do not significantly contribute to hyperconjugation. From Table 2, it is evident that the interaction energy of π donor orbital with π* acceptor orbital is exceptionally high, evidencing an efficient ICT from π → π*. Apart from these π → π* charge transfer, charge transfer is also possible between the lone pair of the O-atom of the carbonyl group and σ* of adjacent C–C bonds, and the lone pair of the S-atom with π* of adjacent C–C bonds.

**Table 2:** Prominent donor–acceptor orbital interaction along with the hyperconjugation energy (E2) in kcal/mol. Representative atom numbers are as shown in the molecular diagram (Figure S5).


Donor → Acceptor orbital	E2 (kcal/mol)	Donor → Acceptor orbital	(E2) )kcal/mol)
C1–C2 (π) → C3–C4 (π*)	15.23	C10–C11 (π) → C6–C7 (π*)	16.71
C1–C2 (π) → C10–C11 (π*)	15.92	C10–C11 (π) → C8–C9 (π*)	15.87
C3–C4 (π) → C1–C2 (π*)	16.77	C22–C24 (π) → C3–C4 (π*)	11.44
C3–C4 (π) → C10–C11 (π*)	15.27	C22–C24 (π) → C26–O27 (π*)	19.78
C3–C 4 (π) → C22–C24 (π*)	15.75	C28–C29 (π) → C31–C 32 (π*)	17.54
C6–C 7 (π) → C8–C 9 (π*)	17.93	C31–C32 (π) → C26–O 27 (π*)	20.21
C6–C 7 (π) → C10–C11 (π*)	15.92	C31–C32 (π) → C28–C 29 (π*)	13.53
C8–C 9 (π) → C6–C7 (π*)	17.06	LP (2) O27 → C24–C 26 (σ*)	20.68
C8–C 9 (π) → C10–C11 (π*)	16.98	LP (2) O27 → C26–C 31 (σ*)	20.28
C10–C11 (π) → C1–C2 (π*)	14.84	LP (2) S 30 → C28–C 29 (π*)	24.65
C10–C11 (π) → C3–C4 (π*)	19.05	LP (2) S 30 → C31–C32 (π*)	20.91

#### Fluorescence spectral analysis

##### Fluorescence in solid state

**Fig. 12:**
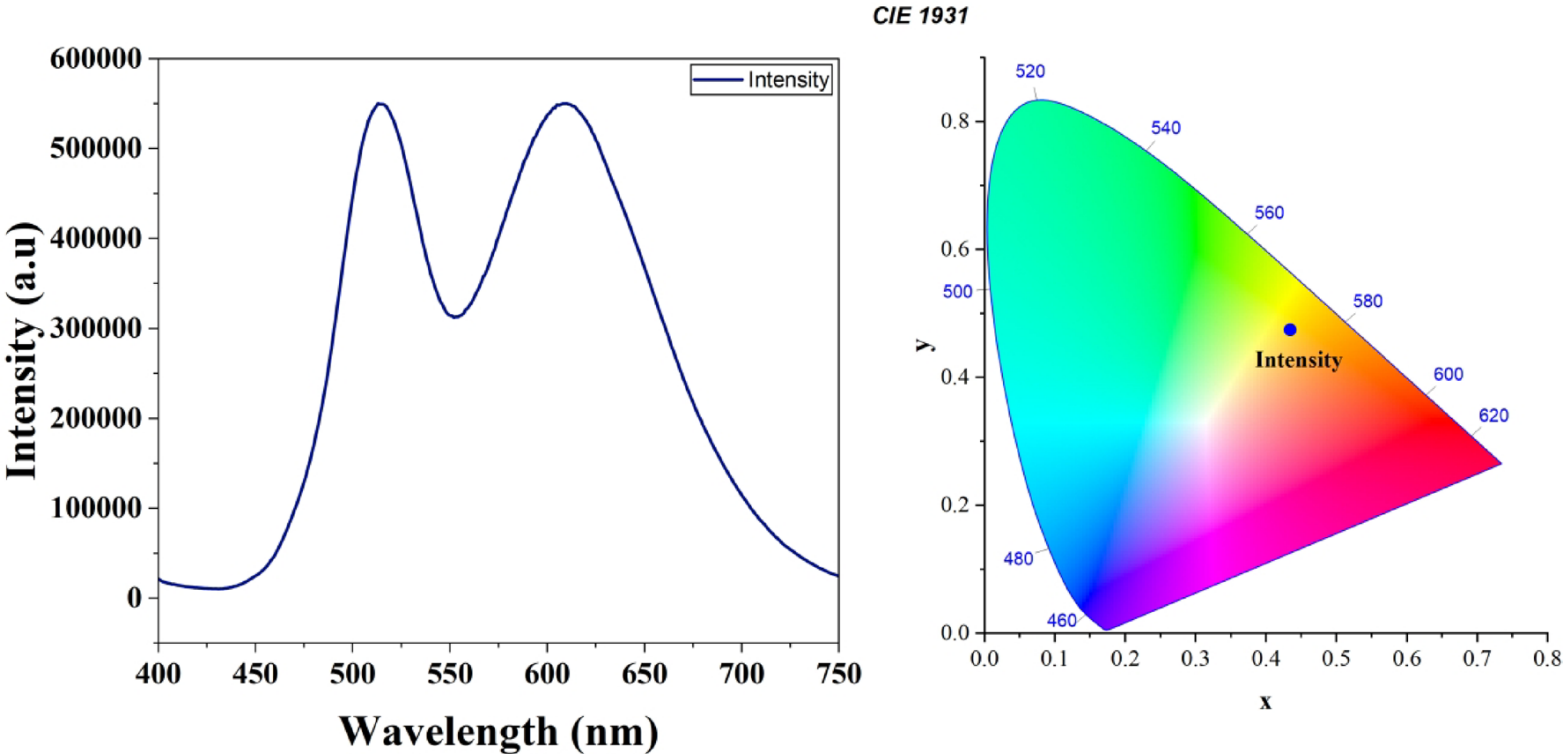
Solid-state fluorescence spectra display dual emission peaks at approximately 510 nm and 610 nm, and the chromaticity diagram of P1 indicating the emission color coordinates.

The solid-state emission of P1 exhibited dual emission peaks near 510 nm and 610 nm, indicating a strong luminescent nature that spans both the orange and green regions (Fig. 12)^31^. Similar dual-emission behaviour has also been observed in thiophene- and naphthalene-based systems, such as (Z)-2 H-naphtho[1,8-bc]thiophenes, naphthalene diimides, and diphenylnaphthalenes, where rigid molecular packing, π–π stacking, and aggregation effects stabilize multiple emissive states. These studies collectively demonstrate that restricted intramolecular motion and intermolecular interactions in fused or substituted aromatic frameworks can generate dual solid-state emission^32–34^. To establish factors such as rigid molecular packing, π–π stacking, and aggregation effects, to explain dual emission behavior, thorough investigation of the crystal packing is required. The physical state of compound P1 (yellowish, non-transparent powder) prevents the single-crystal XRD study. Although powder XRD of the compound was recorded (Figure S6), it was difficult to obtain the accurate crystal information file, and therefore, the crystal packing structure. As discussed above, the DFT calculation reveals that the first excited state of the molecule in absence of solvent environment has twisted structure, which may result in a twisted intramolecular charge transfer (TICT) state. The dual-emission behavior of the molecule is discussed in the following section, on the basis of TICT state. The CIE 1931 plot (Fig. 12) shows that the emission coordinates fall within the yellow-orange region, consistent with the observed emission color. These findings underline the compound’s potential for future use in optoelectronic and luminescent materials.

##### Fluorescence in solution

**Fig. 13:**
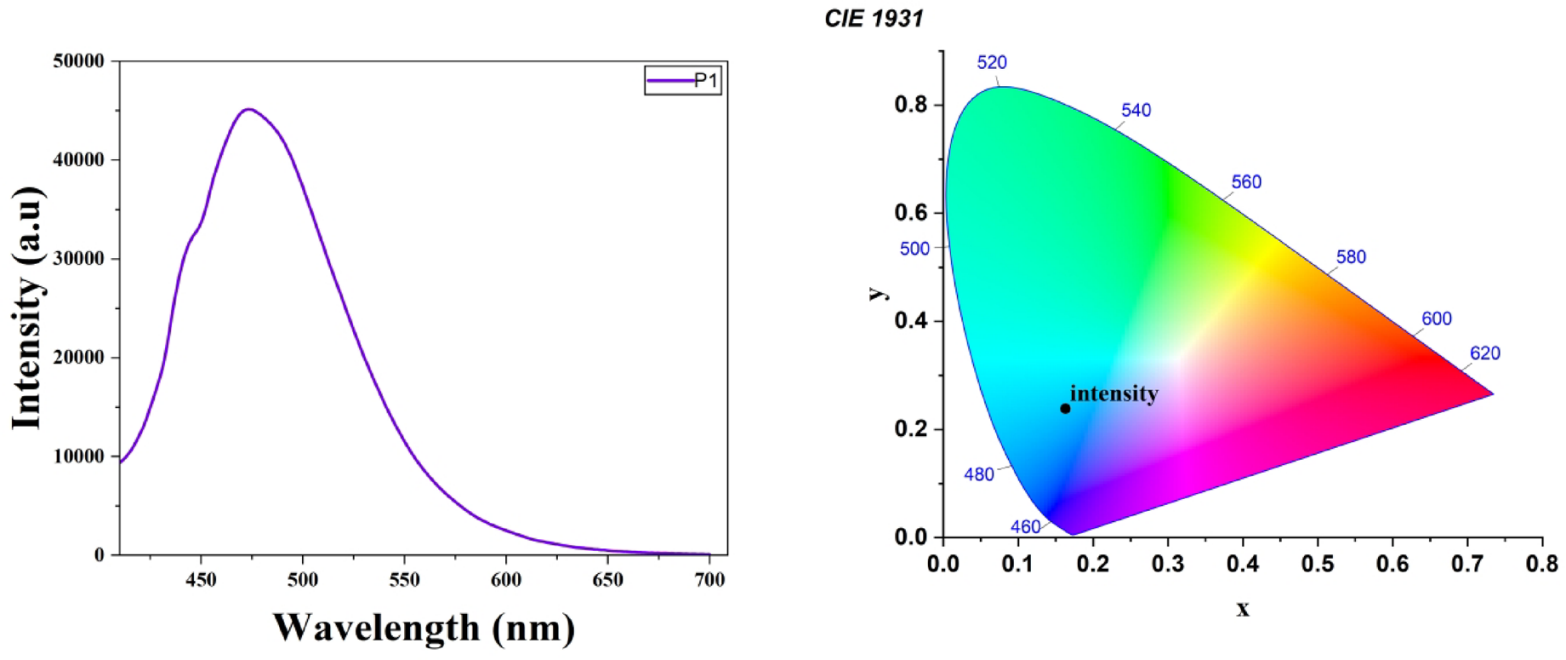
Solution state fluorescence emission spectra of P1 in DMSO display maximum emission at 475 nm and its chromaticity plot indicating the emission color coordinates.

Photoluminescence (PL) spectroscopy, a non-invasive method, was employed to examine the material’s optical emission properties. The photoluminescent behaviour of P1 was investigated in DMSO solution. The emission spectrum (Fig. 13) of P1, recorded with an excitation wavelength of λex = 390 nm, exhibits a broad band spanning 440–650 nm, with a maximum intensity at 475 nm. This characteristic peak indicates emission in the cyan-blue region of the visible spectrum, originating from the extended π-conjugated system and intramolecular charge-transfer processes. The corresponding CIE 1931 chromaticity coordinates fall in the blue-green region, suggesting an observed emission color of aqua to greenish blue. This emission behavior reflects a relatively isolated molecular environment with reduced intermolecular interactions in the liquid phase.

The quantum yield is the ratio of photons emitted to those absorbed, representing the efficiency of the radiative deactivation process. The sample was prepared in DMSO (0.01 mM) at 400 nm, and the quantum yield was calculated using the instrument’s quantum yield calculation software, yielding 63.16%.

##### Theoretical interpretation of emissive properties

To understand the fluorescence behavior of the molecule P1 in the solid state and in DMSO, geometry optimization of the excited states S_1_, S_2_, and S_3_ without and with DMSO was attempted using TD-B3LYP theory with TZVP basis set. All the geometry optimizations were followed by frequency calculation to ensure the absence of negative frequencies. In the solvent-free environment, geometry optimization to the S_2_ state always converges to the S_1_ state structure, whereas in the DMSO environment, the S_3_ state converges to the S_2_ state. As shown in Fig. 9, in the solvent-free environment, the S_2_ state, which was identified as the n-π* state, was 0.05 eV higher in energy compared to the S_1_ state. This small energy barrier between S_2_ and S_1_ may lead to the convergence of geometry to the S_1_ state. Similarly, for the molecule under the DMSO environment, the molecular geometry of S_3_ converges to the S_2_ state. It is important to note that the structure of the molecule P1 is twisted only in the S_1_ state, in the absence of solvent. Other optimized excited state structures, S_3_ in the solvent-free environment, and S_1_ and S_2_ in the DMSO environment, are found to be nearly co-planar, similar to the structure obtained in the S_0_ state.

The calculated value of adiabatic excitation energy, which often resembles the emission transition, for the S_1_ state without solvent is at ~ 572 nm. This is consistent with the experimentally observed solid-state emission band at 610 nm. Under the DMSO environment, the adiabatic excitation energy for the S_1_ state is 491 nm, which is again in very good agreement with the experimentally observed emission band at 475 nm. Both the bands, 572 nm in the solvent-free environment and 491 nm in the DMSO environment shows π-π* characteristic with transition from HOMO→LUMO. As mentioned above, the structure of molecule in the S_1_ state only is twisted, while other excited state structures are nearly co-planar, it is possible that the emission band observed at 510 nm, in the solid state could be due to the locally excited (LE) state which has planar molecular structure whereas the band at 610 nm is due to the twisted intramolecular charge transfer (TICT) state.

**Fig. 14:**
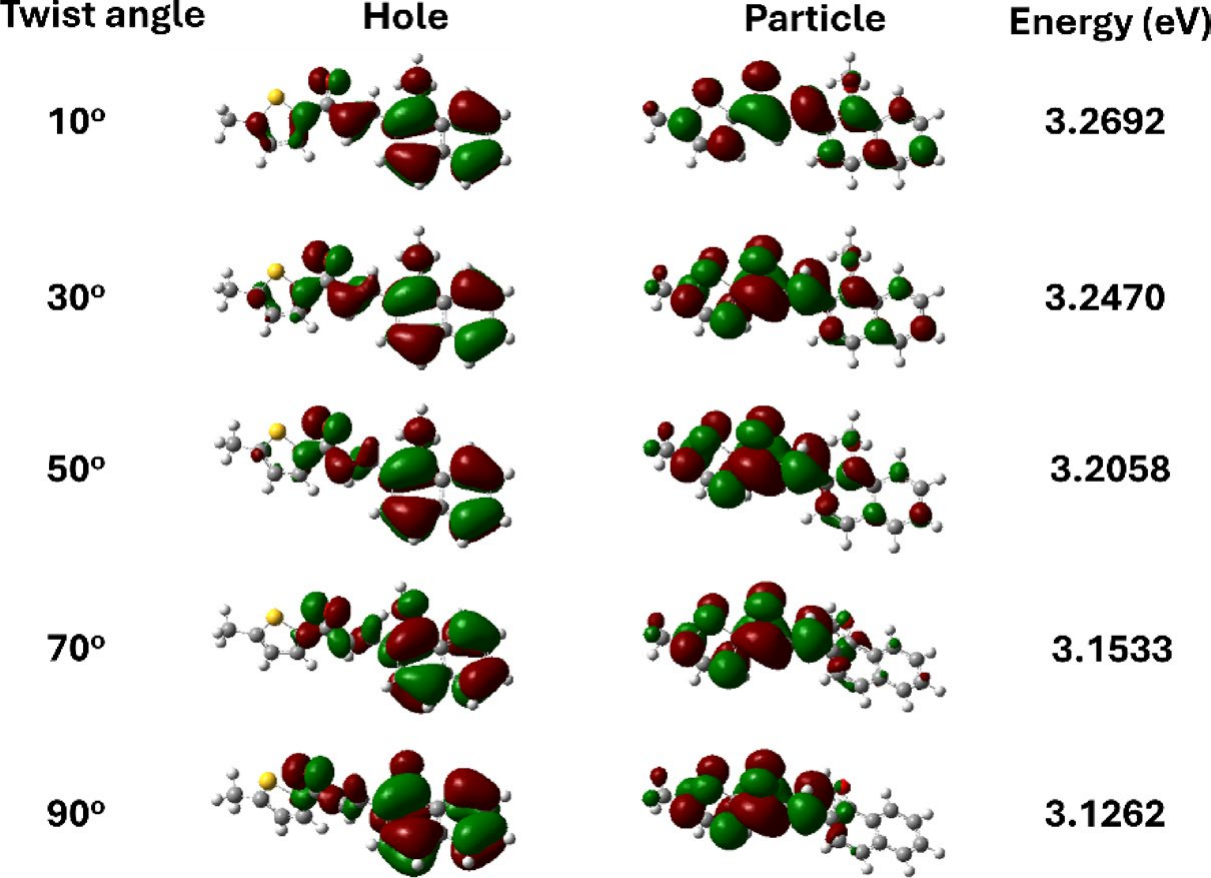
Calculated natural transition orbitals (NTOs) for S_0_→S_1_ for different twist angles between the naphthalene ring and other parts of the molecule along with the calculated excitation energies. Calculations were performed with TD-B3LYP/TZVP and the S_0_ state structure.

To further investigate the dual fluorescence behavior of molecule P1, NTO orbitals S_0_→S_1_ excitation were calculated for different dihedral angles. The obtained Hole and Particle orbitals are shown in Fig. 14. As can be seen, for a dihedral angle 10°, the molecule is nearly planar, and the electrons are delocalized over the entire molecule. As the dihedral angle increases, the energy of the molecule decreases, and the electron cloud tends to be localized on the naphthalene ring in Hole and on the other part of the molecule in particle. The observed orbital picture clearly indicates the planar structure to be the LE state, whereas the twisted structure as TICT state. Radiative transition from both LE and TICT states is possible with TICT transition at a lower energy or higher wavelength. Therefore, the 510 nm emission band in the solid state emission is due to the LE state, and the higher wavelength 610 nm transition is due to the TICT state.

##### Solvent-dependent absorption and emission behaviour of P1 at 20 µM

**Fig. 15:**
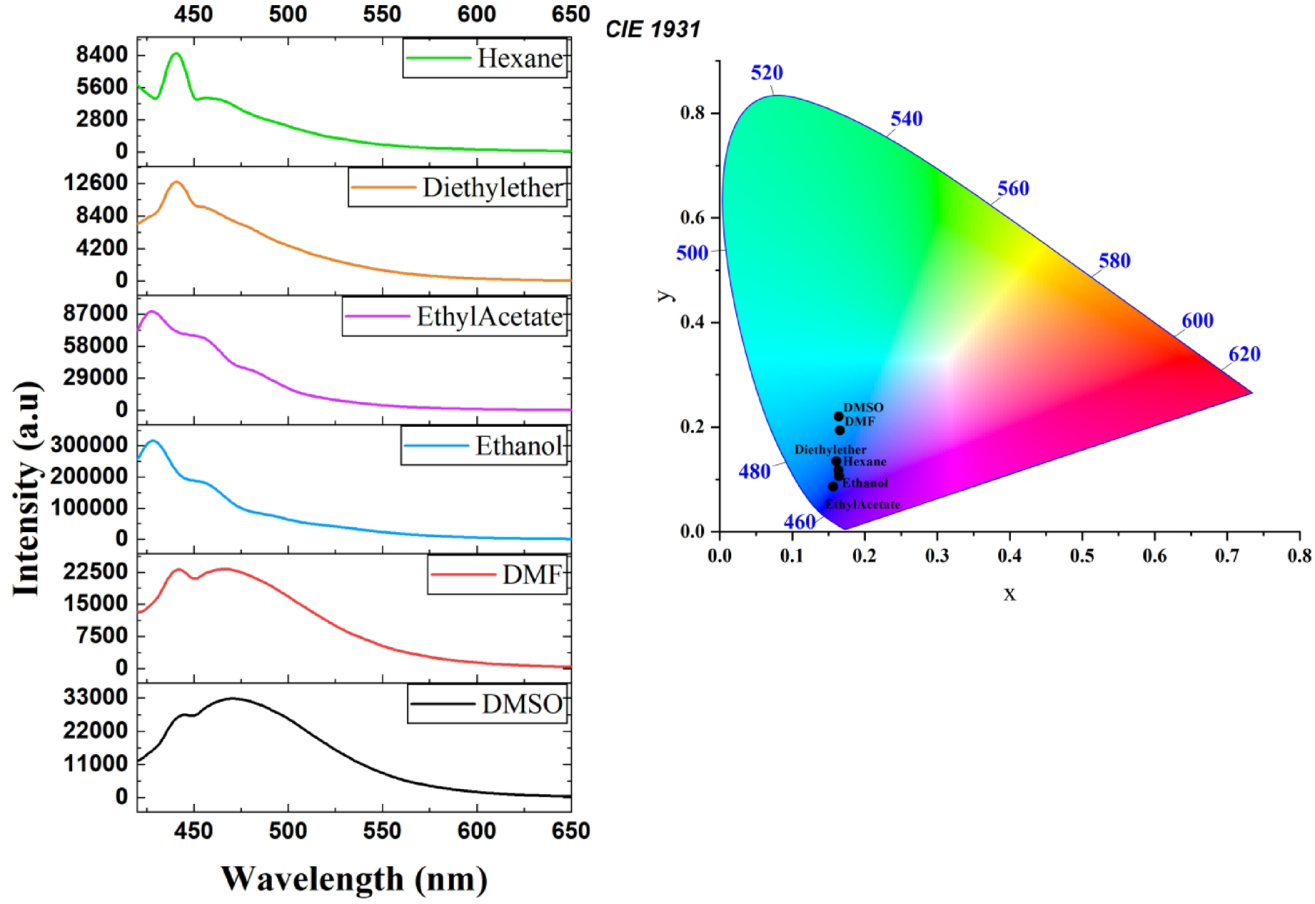
The emission spectra of P1 in different solvent media at 20 µM concentration. And its chromaticity plot.

UV–Vis absorption spectra and PL spectra of P1 were measured at a concentration of 20 µM (Fig. 15a) using a range of solvents with varying polarities, including hexane, diethyl ether, ethyl acetate, ethanol, DMF, and DMSO. The absorption band displays a moderate solvatochromatic shift, whereas the emission maxima exhibit red shifts, accompanied by noticeable intensity changes, as polarity increases, in agreement with intramolecular charge-transfer behavior.

**Table 3:** Orientation polarizability (Δf), maximum absorption wavelength(λ_abs_), maximum emission wavelength (λ_em_), and Stokes’ shift (Δῡ) of P1 in different solvents.

Solvents	∆f	λ_abs_ (nm)	λ_em_ (nm)	Δῡ (cm^− 1^)
Hexane	0.0019	370	441	4351
Diethyl ether	0.168	374	441	4062
Ethyl acetate	0.200	378	427	3036
DMSO	0.263	390	469	4319
DMF	0.275	388	468	4405
Ethanol	0.289	392	429	2200

In Table 3, as solvent polarity increases, the Stokes shift value decreases. This trend can be observed for hexane, diethyl ether, ethyl acetate, and ethanol, but not for DMF and DMSO due to strong dipole–dipole interaction and excited state stabilization^35^.

To study the solvent effect, computed Stokes shifts as wavenumber differences between the absorption maxima and emission maxima,

2 $$\:{\Delta\:}\stackrel{\sim}{{\upnu\:}}={\stackrel{\sim}{{\upnu\:}}}_{\mathrm{Abs}}-{\stackrel{\sim}{{\upnu\:}}}_{\mathrm{Em}},$$

$$\:\mathrm{w}\mathrm{h}\mathrm{e}\mathrm{r}\mathrm{e}\:\mathrm{w}\mathrm{a}\mathrm{v}\mathrm{e}\, \mathrm{n}\mathrm{u}\mathrm{m}\mathrm{b}\mathrm{e}\mathrm{r}\,(\mathrm{c}\mathrm{m})\stackrel{\sim}{{\upnu\:}}=\frac{1}{{\uplambda\:}\:\mathrm{n}\mathrm{m}}$$

The dielectric constants ($\:\epsilon\:)$ and refractive ($\:n$) indices of the solvent can be used to calculate the solvent orientation polarizability $\:{\Delta\:}f$,

3 $$\:{\Delta\:}f=\left(\frac{\epsilon\:-1}{2\epsilon\:+1}\right)-\left(\frac{{n}^{2}-1}{2{n}^{2}+1}\right)$$

A plot of $\:{\Delta\:}\stackrel{\sim}{\nu\:}\:$versus $\:{\Delta\:}f$ yielded a linear correlation,

4 $${\Delta\:}\stackrel{\sim}{\nu\:}=\frac{2({\mu\:}_{e}-{\mu\:}_{g}{)}^{2}}{4\pi\:{\epsilon\:}_{0}hc{a}^{3}}{\Delta\:}f+C$$

where slope

5 $$|m|=\left|\frac{2({\mu\:}_{e}-{\mu\:}_{g}{)}^{2}}{4\pi\:{\epsilon\:}_{0}hc{a}^{3}}\right|=m\frac{2({\Delta\:}\mu\:{)}^{2}}{4\pi\:{\epsilon\:}_{0}hc{a}^{3}}\Rightarrow\:{\Delta\:}\mu\:=\sqrt{\frac{m4\pi\:{\epsilon\:}_{0}hc{a}^{3}}{2}}$$

**Fig. 16:**
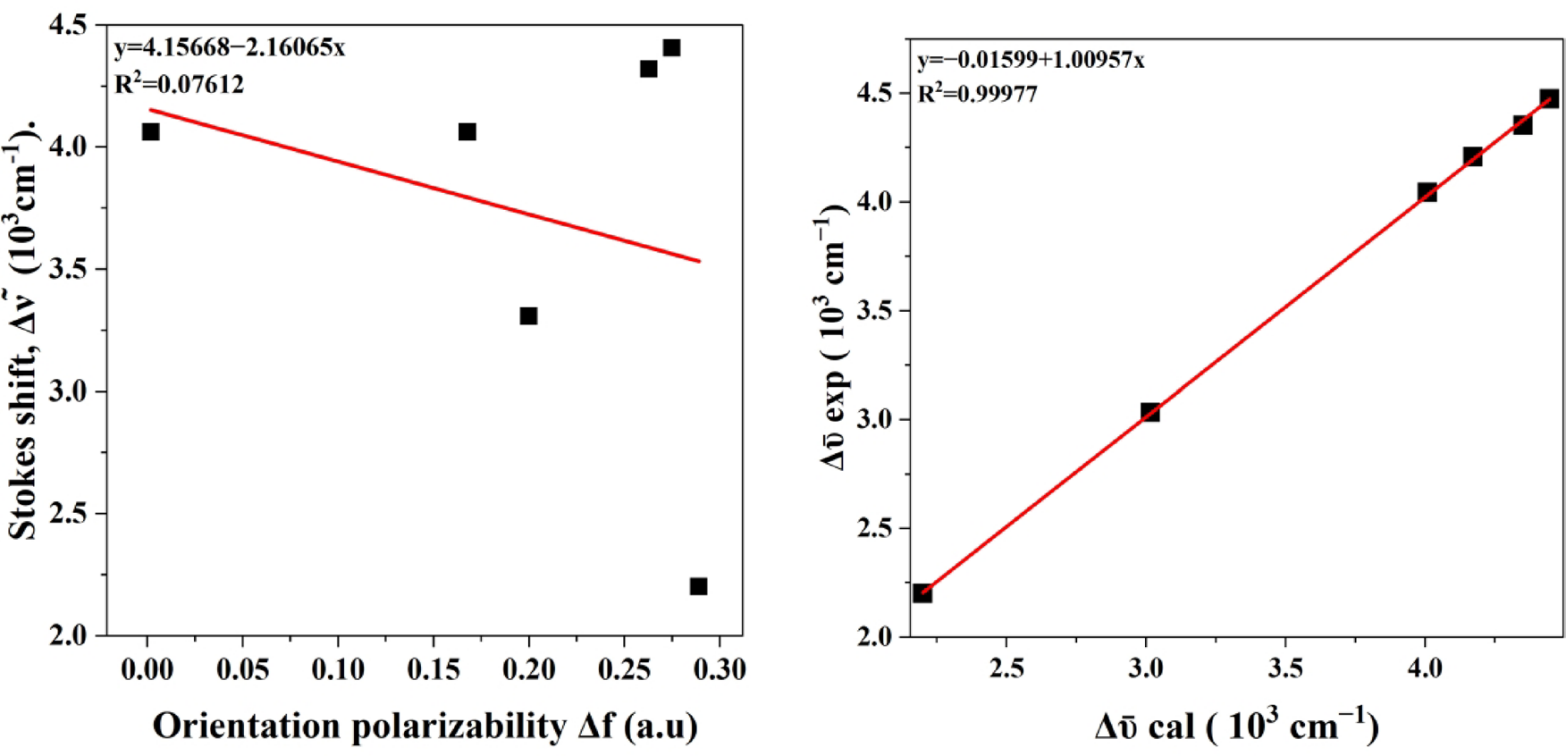
(**a**) Plot of orientation polarizability Δf vs. Stokes shift (Δν ~10^3^cm^−1^). (**b**) Linear relationship between experimental and calculated Δν ~ of P1.

The Lippert–Mataga plot, Δv versus Δf, exhibited poor linearity, with a regression coefficient (R^2^) of 0.07612 (Fig. 16a). This low linear regression coefficient suggests that Lippert–Mataga did not account for other factors, such as ICT and fluorophore hydrogen bonding. In order to address Lippert-Mataga shortages, the P1 solvent interaction and its impact on Stokes’ shifts were described using the LSER^36^, as demonstrated in the following equation.

6 $$(XYZ)s = (XYZ)0 + a\alpha + b\beta + c\pi*$$

where (XYZ)_S_ is the Stokes’ shift, and other parameters (XYZ)_0_ is the Baseline shift (intercept) obtained from the linear multiple regression plot, α is the hydrogen-bond donor acidity of solvent,β is the hydrogen-bond acceptor basicity of solvent, π* is the dipolarity/polarizability of solvent, a, b, c are the regression coefficients. When compared to the Lippert-Mataga plots, the solvent Stokes’ shift demonstrated a regression coefficient (R^2^ = 0.9842), and the results are shown in Table 4. A high R² (0.9842) supports the reliability of the multi-parameter fit, with β’s standard error (± 1.4655) providing confidence in its dominance despite the larger variability. The magnitude of the β coefficient is approximately two to three times larger than that of α and π, highlighting the dominant role of solvent basicity in stabilizing the excited state.

**Table 4:** The fluorescence spectra multilinear regression parameters.

Parameter	Fluroscence	
	Value	Standard error (±)
Intercept	0.65291	0.5626
α	−4.1501	0.4058
β	8.9875	1.4655
π*	−3.0083	0.4853
R^2^	0.9842	

## Conclusion

In this work, a new chalcone derivative, (E)-3-(5-methylthiophen-2-yl)-1-(6 methoxynaphthalen-2-yl)prop-2-en-1-one (P1), was synthesized via a base-catalyzed aldol condensation reaction and characterized using FTIR, ¹H NMR, ¹³C NMR, and mass spectrometry. DFT and TD-DFT supported the experimental findings, indicating solvent-dependent stabilization of excited states and strong ICT character. Photophysical studies revealed dual absorption bands with a bathochromic shift that varied with solvent polarity. Fluorescence studies revealed a single emission in the solution state and dual emissions in the solid state, validating the observed photophysical behavior. Theoretical studies further elucidate the HOMO-LUMO transition, which is consistent with experimental solvatochromatic studies. These results demonstrate that P1 may be a promising candidate for sensing applications and optoelectronic studies due to its strong absorption and emission, as well as its ICT character.

## Electronic Supplementary Material

Below is the link to the electronic supplementary material.

Supplementary Material 1

## Data Availability

Data will be made available on request.
