# Supplementary material for: Synthesis, DFT and photophysical studies of a new thiophene-fused naphthalene chalcone
Source: Sci Rep. 2026 Apr 21;16:18573. doi: 10.1038/s41598-026-49500-4 (PMC13269474; doi:10.1038/s41598-026-49500-4)
Supplement: Supplementary file 1 — Supplementary Material 1 [file 41598_2026_49500_MOESM1_ESM.docx]

**SYNTHESIS, DFT AND PHOTOPHYSICAL STUDIES OF A NEW THIOPHENE-FUSED NAPHTHALENE CHALCONE**

Yogeesh M^1^., Navami Prabhu^1^, Nitinkumar S. Shetty^1^*, Rajeev K. Sinha^2#^

^1^Manipal Institute of Technology, Manipal Academy of Higher Education, Manipal, India. ^2^Department of Physics, Birla Institute of Technology, Mesra, Ranchi - 835215, Jharkhand, India

*****[**nitin.shetty@manipal.edu**](mailto:nitin.shetty@manipal.edu), [**^#^rajeev.sinha@bitmesra.ac.in**](mailto:#rajeev.sinha@bitmesra.ac.in)

**2.3. Analytical and spectral data:**

**
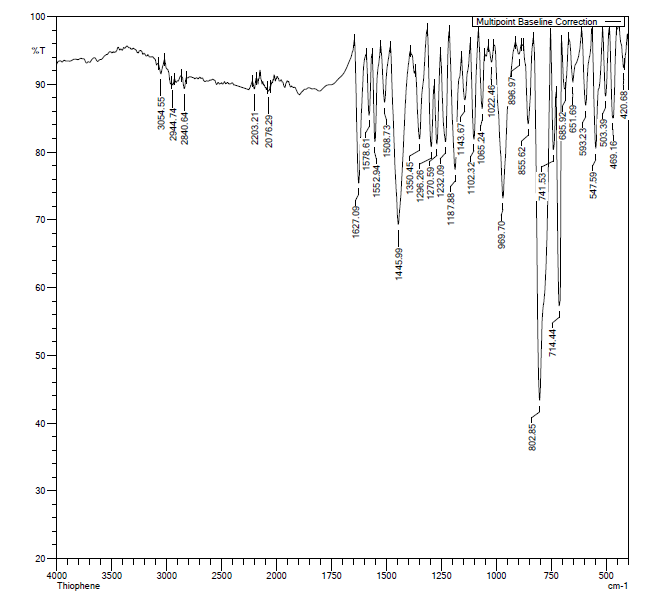
**

**Figure S1:** FTIR spectrum of compound P1 using a Shimadzu FTIR spectrophotometer.

**
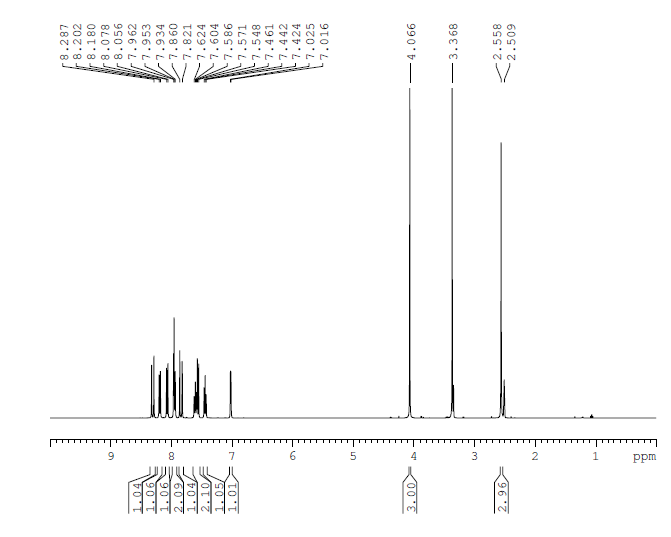
Figure S2:** ^1^H NMR spectrum of compound P1using a Bruker 400 MHz instrument using DMSO as a solvent.


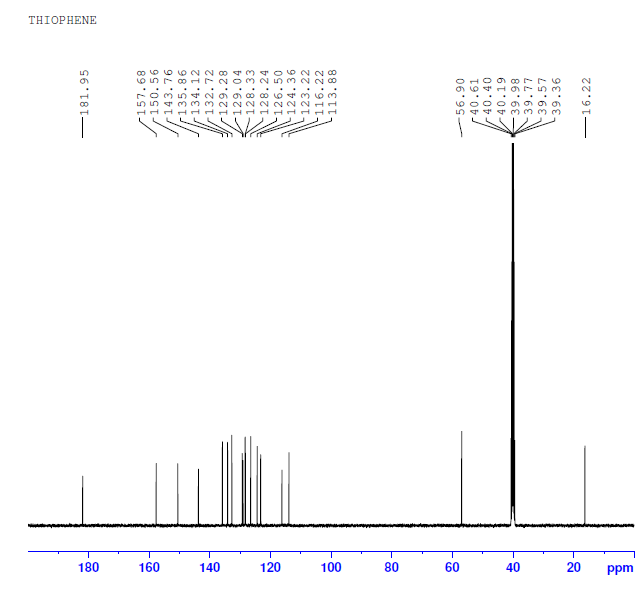


**Figure S3:** ^13^C NMR spectrum of compound P1using a Bruker 100 MHz instrument using DMSO as a solvent.

**
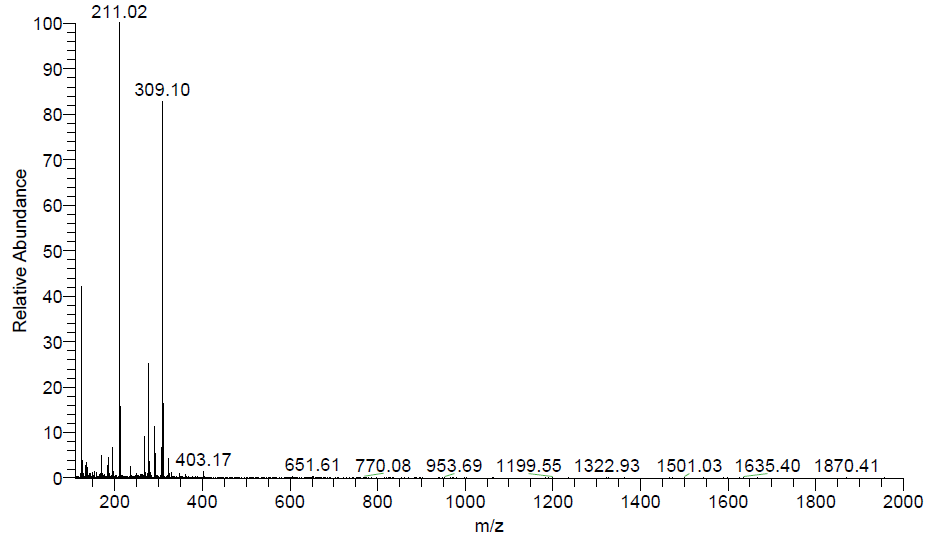
**

**Figure S4:** ESI-MS Spectrum of compound P1.

**3.3.2. Intramolecular charge transfer (ICT):**

**
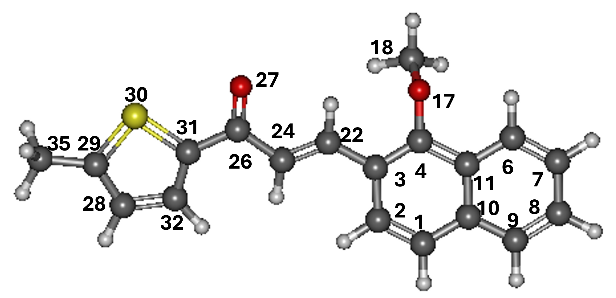
**

**Figure S5:** Structure of molecule with atom numbers used in NBO calculation.

**Table ST1:** Hyperconjugation energy (E(2)) for donor-acceptor natural bond orbital interactions

E(2) E(j)-E(i) F(i,j)

Donor NBO (i) Acceptor NBO (j) kcal/mol a.u. a.u.

======================================================================================

1. BD (1) C 1 – C 2 /471. BD*(1)C 1 – C 10 1.75 1.23 0.041

1. BD (1) C 1 - C 2 /472. BD*(1) C 1 - H 16 0.73 1.13 0.026

1. BD (1) C 1 - C 2 /473. BD*(1) C 2 - C 3 1.73 1.22 0.041

1. BD (1) C 1 - C 2 /474. BD*(1) C 2 - H 15 0.73 1.13 0.026

1. BD (1) C 1 - C 2 /477. BD*(1) C 3 - C 22 3.39 1.19 0.057

1. BD (1) C 1 - C 2 /489. BD*(1) C 9 - C 10 3.48 1.23 0.059

2. BD (2) C 1 - C 2 /476. BD*(2) C 3 - C 4 15.23 0.29 0.061

2. BD (2) C 1 - C 2 /492. BD*(2) C 10 - C 11 15.92 0.29 0.064

3. BD (1) C 1 - C 10 /469. BD*(1) C 1 - C 2 1.68 1.27 0.041

3. BD (1) C 1 - C 10 /474. BD*(1) C 2 - H 15 2.92 1.09 0.051

3. BD (1) C 1 - C 10 /483. BD*(1) C 6 - C 11 3.08 1.19 0.054

3. BD (1) C 1 - C 10 /486. BD*(1) C 8 - C 9 2.02 1.25 0.045

3. BD (1) C 1 - C 10 /489. BD*(1) C 9 - C 10 1.70 1.19 0.040

3. BD (1) C 1 - C 10 /491. BD*(1) C 10 - C 11 1.49 1.18 0.038

4. BD (1) C 1 - H 16 /469. BD*(1) C 1 - C 2 0.56 1.10 0.022

4. BD (1) C 1 - H 16 /473. BD*(1) C 2 - C 3 4.94 1.01 0.063

4. BD (1) C 1 - H 16 /491. BD*(1) C 10 - C 11 4.95 1.02 0.063

5. BD (1) C 2 - C 3 /469. BD*(1) C 1 - C 2 1.67 1.26 0.041

5. BD (1) C 2 - C 3 /472. BD*(1) C 1 - H 16 2.97 1.08 0.051

5. BD (1) C 2 - C 3 /475. BD*(1) C 3 - C 4 1.78 1.21 0.042

5. BD (1) C 2 - C 3 /477. BD*(1) C 3 - C 22 1.51 1.15 0.037

5. BD (1) C 2 - C 3 /479. BD*(1) C 4 - O 17 4.74 1.03 0.062

5. BD (1) C 2 - C 3 /497. BD*(1) C 22 - H 23 1.76 1.11 0.040

6. BD (1) C 2 - H 15 /469. BD*(1) C 1 - C 2 0.53 1.10 0.022

6. BD (1) C 2 - H 15 /471. BD*(1) C 1 - C 10 4.73 1.02 0.062

6. BD (1) C 2 - H 15 /475. BD*(1) C 3 - C 4 4.15 1.05 0.059

7. BD (1) C 3 - C 4 /473. BD*(1) C 2 - C 3 1.69 1.21 0.040

7. BD (1) C 3 - C 4 /474. BD*(1) C 2 - H 15 2.31 1.12 0.045

7. BD (1) C 3 - C 4 /477. BD*(1) C 3 - C 22 1.36 1.17 0.036

7. BD (1) C 3 - C 4 /478. BD*(1) C 4 - C 11 1.66 1.20 0.040

7. BD (1) C 3 - C 4 /483. BD*(1) C 6 - C 11 3.08 1.22 0.055

7. BD (1) C 3 - C 4 /493. BD*(1) O 17 - C 18 0.81 0.98 0.025

7. BD (1) C 3 - C 4 /498. BD*(1) C 22 - C 24 2.36 1.32 0.050

8. BD (2) C 3 - C 4 /470. BD*(2) C 1 - C 2 16.77 0.29 0.064

8. BD (2) C 3 - C 4 /476. BD*(2) C 3 - C 4 0.72 0.29 0.013

8. BD (2) C 3 - C 4 /492. BD*(2) C 10 - C 11 15.27 0.29 0.061

8. BD (2) C 3 - C 4 /493. BD*(1) O 17 - C 18 2.00 0.52 0.031

8. BD (2) C 3 - C 4 /494. BD*(1) C 18 - H 19 0.51 0.62 0.017

8. BD (2) C 3 - C 4 /499. BD*(2) C 22 - C 24 15.75 0.30 0.065

9. BD (1) C 3 - C 22 /469. BD*(1) C 1 - C 2 2.07 1.25 0.046

9. BD (1) C 3 - C 22 /473. BD*(1) C 2 - C 3 1.43 1.16 0.036

9. BD (1) C 3 - C 22 /475. BD*(1) C 3 - C 4 1.54 1.20 0.038

9. BD (1) C 3 - C 22 /478. BD*(1) C 4 - C 11 3.47 1.16 0.057

9. BD (1) C 3 - C 22 /498. BD*(1) C 22 - C 24 1.66 1.28 0.041

9. BD (1) C 3 - C 22 /501. BD*(1) C 24 - C 26 2.89 1.08 0.050

10. BD (1) C 4 - C 11 /475. BD*(1) C 3 - C 4 1.76 1.22 0.041

10. BD (1) C 4 - C 11 /477. BD*(1) C 3 - C 22 3.51 1.15 0.057

10. BD (1) C 4 - C 11 /481. BD*(1) C 6 - C 7 1.96 1.26 0.044

10. BD (1) C 4 - C 11 /483. BD*(1) C 6 - C 11 1.52 1.20 0.038

10. BD (1) C 4 - C 11 /489. BD*(1) C 9 - C 10 2.94 1.20 0.053

10. BD (1) C 4 - C 11 /491. BD*(1) C 10 - C 11 1.48 1.19 0.038

10. BD (1) C 4 - C 11 /493. BD*(1) O 17 - C 18 1.02 0.96 0.028

11. BD (1) C 4 - O 17 /473. BD*(1) C 2 - C 3 2.08 1.36 0.048

11. BD (1) C 4 - O 17 /491. BD*(1) C 10 - C 11 2.04 1.37 0.047

11. BD (1) C 4 - O 17 /494. BD*(1) C 18 - H 19 0.72 1.23 0.027

12. BD (1) H 5 - C 6 /484. BD*(1) C 7 - C 8 4.50 1.03 0.061

12. BD (1) H 5 - C 6 /491. BD*(1) C 10 - C 11 4.89 1.01 0.063

13. BD (1) C 6 - C 7 /478. BD*(1) C 4 - C 11 3.42 1.21 0.058

13. BD (1) C 6 - C 7 /480. BD*(1) H 5 - C 6 0.67 1.14 0.025

13. BD (1) C 6 - C 7 /483. BD*(1) C 6 - C 11 1.73 1.22 0.041

13. BD (1) C 6 - C 7 /484. BD*(1) C 7 - C 8 1.50 1.23 0.038

13. BD (1) C 6 - C 7 /485. BD*(1) C 7 - H 14 0.61 1.13 0.023

13. BD (1) C 6 - C 7 /488. BD*(1) C 8 - H 13 2.33 1.12 0.046

14. BD (2) C 6 - C 7 /487. BD*(2) C 8 - C 9 17.93 0.29 0.064

14. BD (2) C 6 - C 7 /492. BD*(2) C 10 - C 11 15.92 0.28 0.063

15. BD (1) C 6 - C 11 /471. BD*(1) C 1 - C 10 3.04 1.19 0.054

15. BD (1) C 6 - C 11 /475. BD*(1) C 3 - C 4 2.73 1.21 0.052

15. BD (1) C 6 - C 11 /478. BD*(1) C 4 - C 11 1.69 1.17 0.040

15. BD (1) C 6 - C 11 /481. BD*(1) C 6 - C 7 1.47 1.26 0.039

15. BD (1) C 6 - C 11 /485. BD*(1) C 7 - H 14 2.84 1.09 0.050

15. BD (1) C 6 - C 11 /491. BD*(1) C 10 - C 11 1.90 1.18 0.042

16. BD (1) C 7 - C 8 /480. BD*(1) H 5 - C 6 2.81 1.11 0.050

16. BD (1) C 7 - C 8 /481. BD*(1) C 6 - C 7 1.62 1.26 0.040

16. BD (1) C 7 - C 8 /486. BD*(1) C 8 - C 9 1.58 1.26 0.040

16. BD (1) C 7 - C 8 /490. BD*(1) C 9 - H 12 2.97 1.09 0.051

17. BD (1) C 7 - H 14 /483. BD*(1) C 6 - C 11 4.78 1.02 0.062

17. BD (1) C 7 - H 14 /486. BD*(1) C 8 - C 9 3.80 1.08 0.057

18. BD (1) C 8 - C 9 /471. BD*(1) C 1 - C 10 3.58 1.22 0.059

18. BD (1) C 8 - C 9 /484. BD*(1) C 7 - C 8 1.47 1.23 0.038

18. BD (1) C 8 - C 9 /485. BD*(1) C 7 - H 14 2.33 1.13 0.046

18. BD (1) C 8 - C 9 /488. BD*(1) C 8 - H 13 0.60 1.13 0.023

18. BD (1) C 8 - C 9 /489. BD*(1) C 9 - C 10 1.71 1.23 0.041

18. BD (1) C 8 - C 9 /490. BD*(1) C 9 - H 12 0.65 1.12 0.024

19. BD (2) C 8 - C 9 /482. BD*(2) C 6 - C 7 17.06 0.29 0.063

19. BD (2) C 8 - C 9 /492. BD*(2) C 10 - C 11 16.98 0.28 0.065

20. BD (1) C 8 - H 13 /481. BD*(1) C 6 - C 7 3.80 1.09 0.058

20. BD (1) C 8 - H 13 /489. BD*(1) C 9 - C 10 4.68 1.03 0.062

21. BD (1) C 9 - C 10 /469. BD*(1) C 1 - C 2 1.90 1.27 0.044

21. BD (1) C 9 - C 10 /471. BD*(1) C 1 - C 10 1.66 1.19 0.040

21. BD (1) C 9 - C 10 /478. BD*(1) C 4 - C 11 2.87 1.18 0.052

21. BD (1) C 9 - C 10 /486. BD*(1) C 8 - C 9 1.54 1.25 0.039

21. BD (1) C 9 - C 10 /488. BD*(1) C 8 - H 13 2.79 1.10 0.050

21. BD (1) C 9 - C 10 /491. BD*(1) C 10 - C 11 1.65 1.19 0.040

22. BD (1) C 9 - H 12 /484. BD*(1) C 7 - C 8 4.37 1.03 0.060

22. BD (1) C 9 - H 12 /491. BD*(1) C 10 - C 11 5.15 1.02 0.065

23. BD (1) C 10 - C 11 /471. BD*(1) C 1 - C 10 1.36 1.18 0.036

23. BD (1) C 10 - C 11 /472. BD*(1) C 1 - H 16 2.53 1.08 0.047

23. BD (1) C 10 - C 11 /478. BD*(1) C 4 - C 11 1.28 1.17 0.034

23. BD (1) C 10 - C 11 /479. BD*(1) C 4 - O 17 3.84 1.02 0.056

23. BD (1) C 10 - C 11 /480. BD*(1) H 5 - C 6 2.43 1.09 0.046

23. BD (1) C 10 - C 11 /483. BD*(1) C 6 - C 11 1.67 1.18 0.040

23. BD (1) C 10 - C 11 /489. BD*(1) C 9 - C 10 1.36 1.18 0.036

23. BD (1) C 10 - C 11 /490. BD*(1) C 9 - H 12 2.51 1.08 0.047

24. BD (2) C 10 - C 11 /470. BD*(2) C 1 - C 2 14.84 0.27 0.060

24. BD (2) C 10 - C 11 /476. BD*(2) C 3 - C 4 19.05 0.27 0.066

24. BD (2) C 10 - C 11 /482. BD*(2) C 6 - C 7 16.71 0.27 0.064

24. BD (2) C 10 - C 11 /487. BD*(2) C 8 - C 9 15.87 0.27 0.062

25. BD (1) O 17 - C 18 /475. BD*(1) C 3 - C 4 0.65 1.32 0.026

25. BD (1) O 17 - C 18 /476. BD*(2) C 3 - C 4 2.90 0.82 0.047

25. BD (1) O 17 - C 18 /478. BD*(1) C 4 - C 11 0.91 1.28 0.030

26. BD (1) C 18 - H 19 /479. BD*(1) C 4 - O 17 3.90 0.86 0.052

27. BD (1) C 18 - H 20 /493. BD*(1) O 17 - C 18 0.80 0.79 0.022

28. BD (1) C 18 - H 21 /493. BD*(1) O 17 - C 18 0.78 0.79 0.022

29. BD (1) C 22 - H 23 /473. BD*(1) C 2 - C 3 5.18 1.00 0.064

29. BD (1) C 22 - H 23 /498. BD*(1) C 22 - C 24 0.79 1.12 0.027

29. BD (1) C 22 - H 23 /500. BD*(1) C 24 - H 25 6.20 0.91 0.067

29. BD (1) C 22 - H 23 /501. BD*(1) C 24 - C 26 0.56 0.92 0.020

30. BD (1) C 22 - C 24 /475. BD*(1) C 3 - C 4 2.20 1.27 0.047

30. BD (1) C 22 - C 24 /477. BD*(1) C 3 - C 22 1.64 1.21 0.040

30. BD (1) C 22 - C 24 /497. BD*(1) C 22 - H 23 0.78 1.17 0.027

30. BD (1) C 22 - C 24 /500. BD*(1) C 24 - H 25 1.06 1.15 0.031

30. BD (1) C 22 - C 24 /501. BD*(1) C 24 - C 26 1.14 1.16 0.033

30. BD (1) C 22 - C 24 /504. BD*(1) C 26 - C 31 2.02 1.16 0.044

31. BD (2) C 22 - C 24 /476. BD*(2) C 3 - C 4 11.44 0.30 0.054

31. BD (2) C 22 - C 24 /503. BD*(2) C 26 - O 27 19.78 0.29 0.069

32. BD (1) C 24 - H 25 /497. BD*(1) C 22 - H 23 4.37 0.95 0.058

32. BD (1) C 24 - H 25 /498. BD*(1) C 22 - C 24 1.01 1.14 0.030

32. BD (1) C 24 - H 25 /502. BD*(1) C 26 - O 27 3.68 1.11 0.057

33. BD (1) C 24 - C 26 /477. BD*(1) C 3 - C 22 4.79 1.11 0.065

33. BD (1) C 24 - C 26 /498. BD*(1) C 22 - C 24 1.45 1.26 0.038

33. BD (1) C 24 - C 26 /511. BD*(1) S 30 - C 31 3.75 0.84 0.050

34. BD (1) C 26 - O 27 /500. BD*(1) C 24 - H 25 0.91 1.45 0.033

34. BD (1) C 26 - O 27 /501. BD*(1) C 24 - C 26 0.96 1.46 0.034

34. BD (1) C 26 - O 27 /504. BD*(1) C 26 - C 31 0.91 1.46 0.033

34. BD (1) C 26 - O 27 /512. BD*(1) C 31 - C 32 1.88 1.57 0.049

35. BD (2) C 26 - O 27 /499. BD*(2) C 22 - C 24 3.62 0.40 0.035

35. BD (2) C 26 - O 27 /513. BD*(2) C 31 - C 32 5.37 0.37 0.043

36. BD (1) C 26 - C 31 /498. BD*(1) C 22 - C 24 1.94 1.29 0.045

36. BD (1) C 26 - C 31 /502. BD*(1) C 26 - O 27 0.51 1.26 0.023

36. BD (1) C 26 - C 31 /507. BD*(1) C 28 - C 32 1.89 1.18 0.042

36. BD (1) C 26 - C 31 /512. BD*(1) C 31 - C 32 1.92 1.21 0.043

37. BD (1) C 28 - C 29 /504. BD*(1) C 26 - C 31 0.54 1.15 0.022

37. BD (1) C 28 - C 29 /507. BD*(1) C 28 - C 32 1.61 1.23 0.040

37. BD (1) C 28 - C 29 /508. BD*(1) C 28 - H 34 0.85 1.13 0.028

37. BD (1) C 28 - C 29 /510. BD*(1) C 29 - C 35 1.22 1.10 0.033

37. BD (1) C 28 - C 29 /514. BD*(1) C 32 - H 33 3.03 1.14 0.052

38. BD (2) C 28 - C 29 /506. BD*(2) C 28 - C 29 0.51 0.29 0.011

38. BD (2) C 28 - C 29 /513. BD*(2) C 31 - C 32 17.54 0.29 0.066

38. BD (2) C 28 - C 29 /515. BD*(1) C 35 - H 36 2.80 0.63 0.039

38. BD (2) C 28 - C 29 /517. BD*(1) C 35 - H 38 2.79 0.63 0.039

39. BD (1) C 28 - C 32 /504. BD*(1) C 26 - C 31 5.26 1.09 0.069

39. BD (1) C 28 - C 32 /505. BD*(1) C 28 - C 29 1.70 1.22 0.041

39. BD (1) C 28 - C 32 /508. BD*(1) C 28 - H 34 0.55 1.08 0.022

39. BD (1) C 28 - C 32 /510. BD*(1) C 29 - C 35 5.67 1.05 0.069

39. BD (1) C 28 - C 32 /512. BD*(1) C 31 - C 32 1.62 1.21 0.040

40. BD (1) C 28 - H 34 /505. BD*(1) C 28 - C 29 1.05 1.06 0.030

40. BD (1) C 28 - H 34 /509. BD*(1) C 29 - S 30 4.99 0.73 0.054

40. BD (1) C 28 - H 34 /512. BD*(1) C 31 - C 32 2.41 1.06 0.045

41. BD (1) C 29 - S 30 /504. BD*(1) C 26 - C 31 4.12 1.05 0.059

41. BD (1) C 29 - S 30 /508. BD*(1) C 28 - H 34 4.80 1.04 0.063

41. BD (1) C 29 - S 30 /516. BD*(1) C 35 - H 37 1.69 1.00 0.037

42. BD (1) C 29 - C 35 /505. BD*(1) C 28 - C 29 1.46 1.20 0.037

42. BD (1) C 29 - C 35 /507. BD*(1) C 28 - C 32 2.21 1.17 0.045

43. BD (1) S 30 - C 31 /501. BD*(1) C 24 - C 26 3.44 1.04 0.054

43. BD (1) S 30 - C 31 /510. BD*(1) C 29 - C 35 4.99 1.00 0.063

43. BD (1) S 30 - C 31 /514. BD*(1) C 32 - H 33 4.89 1.03 0.064

44. BD (1) C 31 - C 32 /502. BD*(1) C 26 - O 27 1.68 1.32 0.042

44. BD (1) C 31 - C 32 /504. BD*(1) C 26 - C 31 1.41 1.15 0.036

44. BD (1) C 31 - C 32 /507. BD*(1) C 28 - C 32 1.50 1.23 0.038

44. BD (1) C 31 - C 32 /508. BD*(1) C 28 - H 34 3.06 1.13 0.052

44. BD (1) C 31 - C 32 /510. BD*(1) C 29 - C 35 0.73 1.10 0.025

44. BD (1) C 31 - C 32 /514. BD*(1) C 32 - H 33 0.76 1.14 0.026

45. BD (2) C 31 - C 32 /503. BD*(2) C 26 - O 27 20.21 0.30 0.070

45. BD (2) C 31 - C 32 /506. BD*(2) C 28 - C 29 13.53 0.29 0.057

46. BD (1) C 32 - H 33 /505. BD*(1) C 28 - C 29 2.41 1.07 0.045

46. BD (1) C 32 - H 33 /511. BD*(1) S 30 - C 31 5.07 0.73 0.054

46. BD (1) C 32 - H 33 /512. BD*(1) C 31 - C 32 0.86 1.07 0.027

47. BD (1) C 35 - H 36 /505. BD*(1) C 28 - C 29 2.66 1.04 0.047

47. BD (1) C 35 - H 36 /506. BD*(2) C 28 - C 29 3.51 0.53 0.041

48. BD (1) C 35 - H 37 /509. BD*(1) C 29 - S 30 6.34 0.71 0.060

49. BD (1) C 35 - H 38 /505. BD*(1) C 28 - C 29 2.68 1.04 0.047

49. BD (1) C 35 - H 38 /506. BD*(2) C 28 - C 29 3.50 0.53 0.041

76. LP (1) O 17 /475. BD*(1) C 3 - C 4 4.18 1.06 0.060

76. LP (1) O 17 /476. BD*(2) C 3 - C 4 5.41 0.57 0.053

76. LP (1) O 17 /494. BD*(1) C 18 - H 19 2.15 0.90 0.040

76. LP (1) O 17 /496. BD*(1) C 18 - H 21 2.54 0.89 0.043

77. LP (2) O 17 /475. BD*(1) C 3 - C 4 4.45 0.87 0.056

77. LP (2) O 17 /476. BD*(2) C 3 - C 4 2.51 0.37 0.029

77. LP (2) O 17 /478. BD*(1) C 4 - C 11 8.93 0.83 0.078

77. LP (2) O 17 /495. BD*(1) C 18 - H 20 6.70 0.69 0.062

77. LP (2) O 17 /496. BD*(1) C 18 - H 21 3.58 0.69 0.045

78. LP (1) O 27 /501. BD*(1) C 24 - C 26 2.25 1.11 0.045

78. LP (1) O 27 /504. BD*(1) C 26 - C 31 2.82 1.11 0.050

79. LP (2) O 27 /501. BD*(1) C 24 - C 26 20.68 0.66 0.106

79. LP (2) O 27 /504. BD*(1) C 26 - C 31 20.28 0.66 0.104

79. LP (2) O 27 /509. BD*(1) C 29 - S 30 0.83 0.45 0.018

79. LP (2) O 27 /512. BD*(1) C 31 - C 32 0.60 0.77 0.020

80. LP (1) S 30 /505. BD*(1) C 28 - C 29 3.45 1.16 0.056

80. LP (1) S 30 /510. BD*(1) C 29 - C 35 0.59 0.99 0.022

80. LP (1) S 30 /512. BD*(1) C 31 - C 32 3.21 1.15 0.054

81. LP (2) S 30 /506. BD*(2) C 28 - C 29 24.65 0.26 0.072

81. LP (2) S 30 /513. BD*(2) C 31 - C 32 20.91 0.25 0.066

**3.3.2. Fluorescence spectral analysis**

**3.3.2.1. Fluorescence in solid state:**


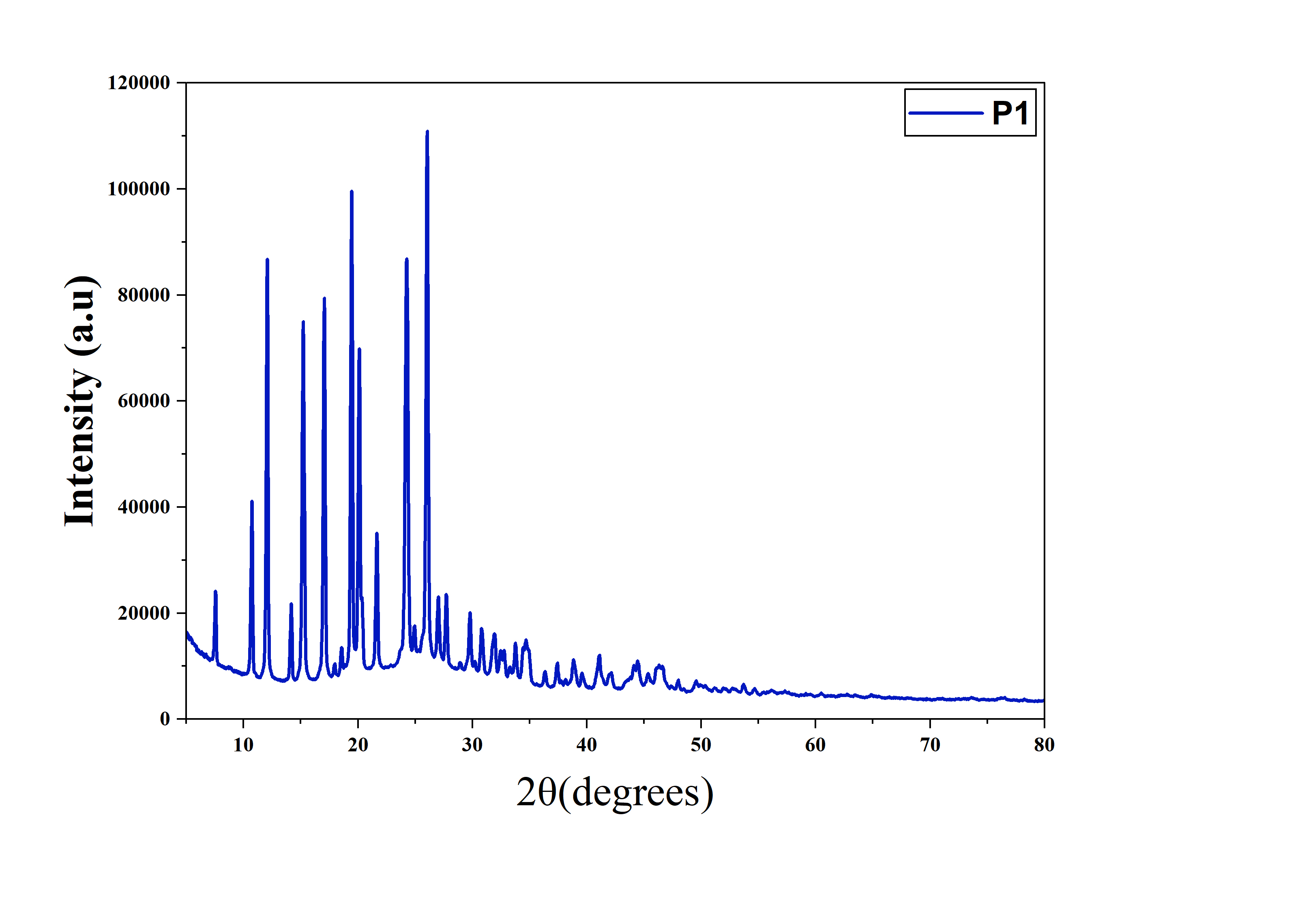


**Figure S6:** Powder X-ray diffraction (XRD) pattern of compound P1, recorded at a scan rate of 2° min⁻¹. The spectrum highlights the crystalline features of P1.
